# Chemical Constituents from the Fruit of *Melia azedarach* and Their Anti-Inflammatory Activity

**DOI:** 10.3390/antiox13111338

**Published:** 2024-10-31

**Authors:** Fan Cao, Jing Chen, Zheng-Tao Lin, Han-Ying Lin, Bin Liu, Zhen-Wei Chen, Xin-Hua Ma, Yong-Hong Zhang

**Affiliations:** Provincial Key Laboratory of Natural Drug Pharmacology, Department of Pharmacy, Fujian Medical University, Fuzhou 350122, China; caofan0101@163.com (F.C.); cj@fjmu.edu.cn (J.C.); linzhengtao0527@163.com (Z.-T.L.); 13705999942@163.com (H.-Y.L.); l_bin_123@163.com (B.L.); chenyi1314170@163.com (Z.-W.C.)

**Keywords:** *Melia azedarach*, meliaceae, limonoids, anti-inflammatory, NF-κB

## Abstract

Phytochemical investigations of *Melia azedarach* fruits have led to the isolation of a novel tirucallane triterpenoid (**1**), four new limonoids (**2**–**5**), and four known limonoids (**6**–**9**). Their structures were clarified by comprehensive spectroscopic and spectrometric analyses. The anti-inflammatory activities of isolated compounds were assessed in vitro. Compound **2** exhibited the most potent anti-inflammatory effect, with an IC_50_ value of 22.04 μM. Additionally, compound **2** attenuated LPS-induced reactive oxygen species (ROS) production and reduced the levels of inflammatory mediators IL-6 and TNF-*α*. A mechanistic study revealed that limonoid **2** suppresses the expression of iNOS and JAK2 and is implicated in the modulation of the NF-κB signaling cascade, which reveals its anti-inflammatory actions.

## 1. Introduction

Reactive oxygen species (ROS), natural by-products of oxygen metabolism, are critical in cell signaling and homeostasis [1]. However, excessive ROS generation during various infections and pathological states can lead to protein and nucleic acid oxidation, affecting toxic inflammatory effects on cellular structures [2]. These highly reactive, electron-deficient radicals cause oxidative damage to cellular membranes, DNA, and proteins. Persistent oxidative injury activates intracellular signaling cascades, leading to a chronic systemic inflammatory response, exacerbating conditions such as cardiovascular disorders and cancer [3,4].

Inflammation is a significant risk factor for numerous disorders, with macrophages playing a vital role as primary immune cells defending against pathogens, such as bacteria and viruses [5]. During inflammation, macrophages overproduce inducible nitric oxide synthase and pro-inflammatory factors such as IL-6 and TNF-α [6]. Excessive production of these mediators can worsen conditions such as allergies, autoimmune diseases, cancers, and metabolic syndromes [7,8]. The nuclear factor-κB (NF-κB) pathway mediates the induction of NO, IL-6, TNF-α, and other pro-inflammatory cytokines in mononuclear/macrophages, contributing to the amplification and spread of inflammatory responses [9].

Lipopolysaccharides (LPS), components of the outer cell walls of Gram-negative bacteria, trigger host inflammatory responses by increasing the production of chemokines, cytokines, and pro-inflammatory [10] mediators. LPS-exposed macrophages stimulate cytokine and chemokine production during microbial infections, triggering further inflammatory events. Therefore, suppressing macrophage activation by LPS represents a crucial strategy in targeting inflammatory diseases. Regulation of inflammatory mediators like NF-κB, JAK2, and iNOS, as well as pro-inflammatory factors like NO, IL-6, and TNF-α, could serve as potential therapeutic approaches to treat inflammatory damage [11,12,13,14].

*Melia azedarach* L. (Meliaceae) is native to China and other Southeast Asian countries [15]. The bark and fruit of *Melia azedarach* are traditionally used for insecticidal, analgesic, and dermatological applications [16]. Various constituents, including limonene, triterpenes, and steroids [17,18,19,20,21,22,23,24], have been separated from different parts of the plant, with limonenes demonstrating notable antibacterial [25], cytotoxic [26], food repellent [27], and insecticidal activities [28]. Despite these properties, the anti-inflammatory effects and mechanisms of *M. azedarach* remain poorly understood.

This study utilized LPS-stimulated macrophages to investigate the anti-inflammatory properties of compounds isolated from *M. azedarach* fruit. A new tirucallane triterpenoid (**1**) and four new limonoids (**2**–**5**), along with four known limonoids (**6**–**9**), were isolated and characterized. Compound **2** exhibited the most significant inhibitory effect on nitric oxide production, as demonstrated by the Griess assay. Further cellular assays targeting TNF-α, IL-6, ROS, NF-κB, eNOS, and JAK2 signaling pathways elucidated the anti-inflammatory potential of compound **2**, indicating its action primarily through the NF-κB and JAK2 pathways.

## 2. Material and Methods

### 2.1. General Experimental Procedure

CD spectra were acquired using a Chirascan spectropolarimeter (Applied Photophysics, Surrey, UK). Optical rotation was determined with a JASCO P-1020 rotameter (Tokyo, Japan). Nuclear Magnetic Resonance spectra were recorded on a Bruker ARX-600 spectrometer (Rheinstetten, Germany). HR-ESI-MS was performed using an Agilent 6530B TOF mass spectrometer (Agilent Technologies, Santa Clara, CA, USA). Compounds werepurified using HPLC with a C_18_ column (YMCPackODSA, Waters Company, Milford, MA, USA).

### 2.2. Plant Materials

The fruits of *Melia azedarach* were gathered in November 2021 from Minhou County, Fuzhou City, Fujian Province, China. The plant specimens were authenticated by Professor Dongmei Shi from Fujian Medical University, and the sample voucher (No. 20211112) has been stored in the School of Pharmacy at the same university.

### 2.3. Extraction and Separation

Approximately 23.7 kg of dried fruits of *M. azedarach* were extracted with MeOH three times weekly. After concentrating the combined filtrates, a total of 1.8 kg of crude extract was obtained. This extract was subsequently dissolved in water and sequentially partitioned with hexane, CH_2_Cl_2_, EtOAc, and n-butanol to fractionate the various chemical constituents.

The CH_2_Cl_2_ fraction (0.9 kg) was fractionated over silica gel columns, PE–ethyl acetate (10:1 to 0:2) to afford night-major portions (Fr. 1-9). Fr. 1 (9.8 g) was isolated on an ODS gel column (40%, 60%, 80%, and 100% MeOH/H_2_O), followed by Sephadex LH-20 (MeOH-CH_2_Cl_2_) and HPLC (CH_3_CN/H_2_O) to give compound **1** (15.6 mg). Fr.3 (25.0 g) was partitioned via ODS columns (40%, 60%, 80%, and 100% MeOH/H_2_O) and subsequently purified using SephadexLH-20 (MeOH-CH_2_Cl_2_) and HPLC (CH_3_CN/H_2_O) to provide compounds **2** (21.0 mg), **6** (25.7 mg), and **7** (25.7 mg). Fr.4 (12.0 g) was isolated using an ODS gel column (40:60 to 100:0, MeOH/H_2_O), purified using SephadexLH-20 (90:10, MeOH-CH_2_Cl_2_), and fractionated by HPLC (58:42, MeOH/H_2_O) to give compounds **8** (9.4 mg) and **9** (7.9 mg). Fr.5 (20.0 g) was isolated using an ODS gel column (30:70 to 100:0, MeOH/H_2_O) and four fractions (Fr. 5-1 to 5-4). Separation of Fr. 5-2 (6.5 g) on a SephadexLH-20 column (MeOH), followed by HPLC (58:42, MeOH/H_2_O), afforded compounds **3** (8.5 mg) and **4** (5.7 mg). Fr. 5-3 (8.0 g) was isolated using Sephadex LH-20 (90:10, MeOH-CH_2_Cl_2_) and HPLC (CH_3_CN/H_2_O, 47:53) to obtain compound **5** (11.1 mg).

Compound **1**. Colorless powder; [*α*]^25^_D_: +439° (*c*0.10, MeOH); IR (KBr)*ν*_max_: 3444, 2951, 1705, 1625, 1450, 1384, 1142 cm^−1^; NMR data: see Table 1 and Table 2, Figures S3–S8; HRESIMS *m*/*z* 511.3690 [M + Na]^+^ (Figure S9) (calc. for C_30_H_48_O_5_Na^+^ as 511.3394).

Compound **2**. Colorless powder; [*α*]^25^_D_: +1° (*c*0.10, MeOH); IR (KBr) *ν*_max_: 3446, 2923, 1714, 1629, 1384, 1270, 1144 cm^−1^; NMR data (Table 1 and Table 2, Figures S10–S15); HRESIMS *m*/*z* 589.2812 [M + H]^+^ (Figure S16) (calc. for C_35_H_41_O_8_ as 589.2796).

Compound **3**. **A**morphous powder; [*α*]^21^_D_: +59.4° (*c*0.20, MeOH); IR (KBr) *ν*_max_: 3443, 2923, 1696, 1629, 1460, 1384, 1247 cm^−1^; NMR data (Table 1 and Table 2, Figures S17–S22); HRESIMS *m*/*z* 671.2797 [M + Na]^+^ (Figure S23) (calc. for C_37_H_44_O_10_Na^+^ as 671.2827).

Compound **4**. **A**morphous powder; [*α*]^21^_D_: +69.3° (*c*0.20, MeOH); IR (KBr) *ν*_max_: 3438, 2975, 1700, 1629, 1461, 1384, 1256, 1082 cm^−1^; NMR data (Table 1 and Table 2, Figures S24–S29); HRESIMS *m*/*z* 671.2873 [M + Na]^+^ (Figure S30) (calc. for C_37_H_44_O_10_Na^+^ as 671.2827).

Compound **5**. Colorless powder; [*α*]^21^_D_: −59.4° (*c*0.20, MeOH); IR (KBr) *ν*_max_: 3452, 2928, 1738, 1634, 1384, 1251, 1049 cm^−1^; NMR data (Table 1 and Table 2, Figures S31–S36); HRESIMS *m*/*z* 653.2935 [M + Na]^+^ (Figure S37) (calc. for C_34_H_46_NaO_11_ as 653.2932).

### 2.4. Cell Culture

Mouse macrophages RAW264.7 were procured from the Shanghai Model Culture Preservation Centre, Chinese Academy of Sciences. The cells were incubated on DMEM media enriched by 10% FBS and incubated in a CO_2_ incubator at 37 °C with 5% humidity.

### 2.5. Cell Viability Test

To evaluate cell viability, all isolated compounds from the fruit of *M*. *azedarach* were tested using the SRB assay in an LPS-mediated RAW264.7 cell model [29]. RAW264.7 macrophages were seeded into 96-well plates and cultured for 24 h prior to treatment. Limonoids **1**–**9** were dissolved in DMSO, diluted six-fold with fresh medium, and administered to each well for 24 h. Absorbance was measured at 515 nm using an ELISA reader (BioTek, Winooski, VT, USA).

### 2.6. Anti-Inflammatory Effect Test

The anti-inflammatory activity of isolated compounds was assessed by measuring NO production in macrophages. Cells were prepared as described in the cell viability assay. Limonoids **1**–**9** were treated with the cells for 4 h, followed by a 24 h LPS treatment. NO production in the cell supernatant was determined using the Griess reaction [30]. An equal volume of cell culture supernatant was combined with the Griess reagent, and the absorbance was determined at 540 nm to calculate the IC_50_ values.

### 2.7. Flow Cytometry Test

Cells were pre-treated and seeded in a 6-well plate at a density of 2 × 10^5^ cells in each well and cultivated for 24 h. To detect *ROS* levels, DCFH-DA was added to the medium (20 min, 1 μM) after the incubation period with limonoid 2 [31]. Cells were then washed three times with serum-free medium, and fluorescence signals were collected using a FACScan flow cytometer (Becton Dickinson, Franklin Lakes, NJ, USA).

### 2.8. Cytokine Detection

Cytokine levels of TNF-α and IL-6 in limonoid **2**-treated samples were quantified using enzyme-assisted immunosorbent assays [32]. RAW264.7 cells were incubated in 96-well plates for 24 h. After a 4 h pre-treatment period, cells were stimulated with LPS at a concentration of 2.5 µg/mL for an additional 24 h. The concentrations of IL-6 and TNF-α in the supernatant were then determined with a commercially available ELISA kit.

### 2.9. Western Blotting Analysis

RAW264.7 macrophages were pre-treated with limonoid **2** for 4 h and subsequently mediated by 2.5 µg/mL LPS for 24 h. Cells were lysed using a buffer containing a protease inhibitor cocktail, and total protein was quantified with BCA protein assay kits. The protein was isolated by 10% SDS-PAGE and transferred to a PVDF membrane. Membranes were stopped for 1 h at room temperature with 5% skimmed milk and 0.1% Tween-20 in triple-buffered saline. Overnight culture was conducted at 4 °C using primary antibodies (1:1000 dilution) targeting p65, p-IκBα, p-IKK, p-JAK2, p-STAT3, iNOS, eNOS, and β-actin (Table S1). After washing the membrane with TBST, it was incubated with HRP-labeled secondary antibody for 30 min at room temperature. Immunoreactive bands were detected with the ECL test kit (Supplementary S1).

### 2.10. Immunofluorescence Assay

Immunofluorescence staining was performed to assess whether limonoid 2 affects the nuclear translocation of the p65 subunit. Macrophages were seeded on *confocal dishes* and incubated overnight. Following the incubation period with limonoid **2**, cells were stained with monoclonal primary antibodies and fluorescently labeled secondary antibodies diluted to 1:500 and 1:100, respectively (Table S1) [33]. A Leica TCS SP5 laser confocal microscope (Leica, Wetzlar, Germany) was used for imaging, and image processing was performed using ImageJ software v1.53 (Supplementary S2).

### 2.11. Statistical Analyses

Data are presented as mean ± standard error. The band intensities of the Western blots were quantified using Image Lab 3.0 software. For the calculation of IC_50_, we put the data through the non-linear fitting process of Prism 8 software to obtain the result. All statistical analyses, including *t*-tests or one-way ANOVA to evaluate significant differences, were performed using Prism 8 software. Statistical significance was taken as *p* < 0.05.

## 3. Results

### 3.1. Chemical Structural Determination of Compounds ***1***–***5***

**Figure 1:**
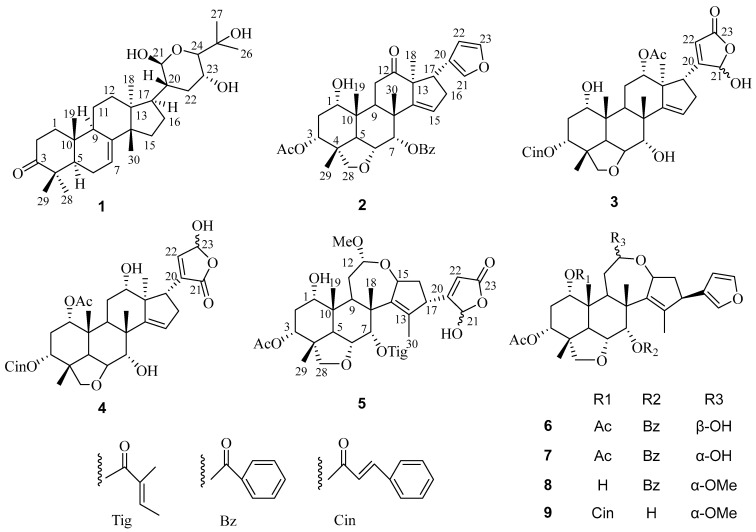
The chemical structures of compounds **1**–**9**.

**Figure 2:**
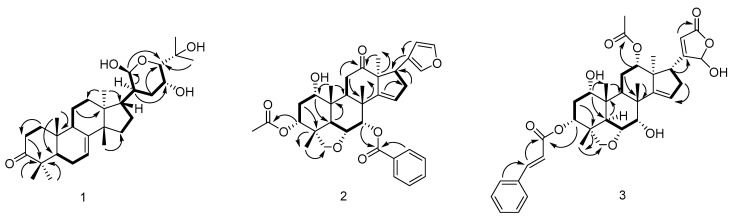

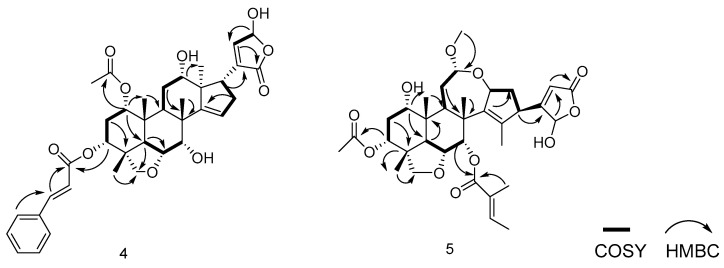
Partial ^1^H-^1^H COSY and HMBC correlation for compounds **1**–**5**.

Compound **1** was separated as a white powder. HRESIMS of compound **1** revealed a conjugated molecular ion peak at *m/z* 511.3690 [M + Na]^+^, consistent with the calculated mass of 511.3394 and confirming the molecular formula C_30_H_48_O_5_. The IR spectroscopy displayed absorption bands characterized by hydroxyl (3444 cm^−1^) and carbonyl (1705 cm^−1^) moieties. The ^1^H- and ^13^C-NMR spectra exhibited signals typical of tirucallane triterpenoids [34]. Comparison with the IR and NMR spectra of bourjotinolone A [35] revealed a close similarity, with the notable difference of an additional hydroxyl group at the C-21 position. This modification was further confirmed through comprehensive analysis using ^13^C NMR and DEPT spectra, where the oxymethine moiety was detected at δ_C_ 101.8 (C-21). Complete assignment of all proton and carbon resonances for compound **1** was accomplished with HMQC, HMBC, and COSY experiments. Specifically, the HMBC correlations from proton H-20 to carbon C-21 (δ_C_ 101.8) provided definitive evidence of the hydroxyl group at the C-21 position (Figure 1). The HMBC correlation of H-21 (δ_H_ 5.48) to C-24 (δ_C_ 86.5), C-20 (δ_C_ 47.1); H-20 (2.03, m, 1H) to C-22 (δ_C_ 33.6), C-23 (δ_C_ 76.7); H-24 (δ_H_ 3.37, 1H, d, *J* = 5.2 Hz)/C-25 (71.1), C-23 (76.7) also demonstrates the existence of a six-membered cycle (Figure 2). In addition, the HMBC correlation of H-17 (δ_H_ 1.85, 1H, m) and C-20 (δ_C_ 47.1), C-21 (δ_C_ 101.8), and C-15 (δ_C_ 34.4) established a link between the tetracyclic portion and the hydroxyl ring, and the relative conformations of **1** were established based on biological genetic concerns and interpretations of NOE data. The core structure of compound **1**, comprising the basic tertiary rings A, B, C, and D, is conformationally identical to those of bourjotinolone A, sharing identical backbones. The NOE correlation of H_3_-30 with H-11 and H_3_-30 with H_3_-19 indicate that these protons are coplanar and assume a *β*-orientation, aligning with previously reported data for bourjotinolone A. Additionally, interactions between H-5 with H_3_-28 and H-20 with H-21 establish the α-orientations of H-5, H-21, H_3_-28, and H-20 (Figure S1). The absolute configuration of compound **1** was verified through comparative experiments and ECD calculations. A systematic conformational search, geometry optimization, and TDDFT/ECD analysis using Gaussian16 [36,37] software confirmed this configuration. The theoretical ECD spectrum of compound **1** matched the experimental (Figure S2) data, allowing the absolute configurations to be precisely defined as 5*R*, 9*R*, 10*R*, 13*S*, 14*S*, 17*R*, 20*S*, 21*R*, 23*R*, and 24*S*. Thus, compound **1** is confidently identified as 21*β*-hydroxy bourjotinolone A.

Compound **2** showed an [M + H]^+^ ion peak in the positive ion mode of HRESIMS at *m/z* 589.2812 (C_35_H_41_O_8_, calcd589.2796), which corresponds to the molecular formula C_35_H_40_O_8_. The IR spectra of compound **2** displayed characteristic absorption bands for hydroxyl (3446 cm^−1^), ester carbonyl (C=O at 1714 cm⁻^1^), additional carbonyl (1629 cm^−1^), and furan (807 cm^−1^) moieties. The ^1^H and ^13^C NMR data of **2** revealed the existence of four tertiary Me moieties (*δ*_H_ 0.97, 0.98, 1.17, and 1.18), one Ac Me moiety (*δ*_H_ 1.82), one benzoyl moiety (δ_H_ 8.05, dd7.6, 1.3; 7.39, t7.6; 7.53, t7.5), one CH_2_-O group (δ_H_ 3.18, d7.8, and 3.46, d7.8; δ_C_ 78.1), three CH-O moieties (δ_H_ 3.59, t 2.8; 5.08, t 2.7; and 5.90, d 3.0), one vinyl CH moiety (δ_H_ 5.73, t 2.5; δ_C_ 123.8), one carbonyl group (δ_C_213.8), and a *β*-substituted furan ring (δ_H_ 7.25, t1.6; 6.48, d1.6; 7.27, s; δ_C_ 124.6, 142.4, 112.6, and 141.0) [38], indicating that **2** shares a nimbidinin-type backbone structure [39] with Ac and benzoyl moieties at C-3 and C-7, respectively (Figure 1). HMBC correlation of H-3 (δ_H_ 5.08, t 2.7) with AcO-3 (δ_C_169.2), of H-7 (δ_H_ 5.90, d 3.0) to C-1′ (δ_C_ 165.1), and of H-9 (δ_H_ 3.58, dd 5.5,7.3) and H-11(δ_H_ 2.54, m, and 2.34, m), and of Me-18 (δ_H_ 0.97) with C-12 (δ_C_ 213.8), and the NOE correlation of the ^1^Hsignal of H-3 (δ_H_ 5.08, t 2.7), Me-29 (δ_H_ 1.17), Me-19 (δ_H_ 0.98), Me-30 (δ_H_ 1.18), and H-7, and of Me-30 and H-17 (δ_H_ 3.46, m) of **2** supported the proposed structure (Figure 2). The relative configurations of compound **2** were ascertained using NOESY correlations alongside a comparison of the NMR data with that of nimbidinin (Figure S1). The computed ECD spectra (Figure S2) align well with experimental observations, confirming the absolute configurations of 1*S*, 3*R*, 4*R*, 5*R*, 6*R*, 7*S*, 8*R*, 9*S*, 10*R*, 13*S*, and 17*R*. As a result, the structure of **2** has been elucidated as 3-acetyl-7-benzoylnimbidinin.

Compound **3** was separated as an amorphous powder. HRESIMS analysis of compound **3** displayed a [M + Na]^+^ peak at *m*/*z* 671.2797, which is in close agreement with the calculated value (671.2827) for the molecular formula C_37_H_44_O_10_. The IR spectroscopy indicated the existence of OH at 3443 cm^−1^, C=O at 1696 cm^−1^, and double bond (1629 cm^−1^) moieties. The ^1^H NMR and COSY spectra displayed similarities to those of compound **14** [40] with features including four methyl groups (*δ*_H_ 0.95, s, 3H; *δ*_H_ 1.00, s, 3H; *δ*_H_ 1.15, s, 3H; and *δ*_H_ 1.09, s, 3H), an acetyl methyl (*δ*_H_ 2.03, s, 3H), and a cinnamoyl (*δ*_H_ 7.48–7.38, m, 5H; *δ*_H_ 7.72, d, *J* = 16.0, H-3′; *δ*_H_ 6.40, d, *J* = 16.0, H-2′) group. The distinction between compound **3** and compound **14** is notable in the E-ring, where compound **3** shows two broad singlets at 5.88 (H-21) and 5.85 (H-22) for a *γ*-hydroxylbutyrolactone [19], in contrast to the furan chain signals found in compound **14** [40]. The ^13^C NMR spectra of **3** confirmed the presence of hemiketal carbon (*δ_C_* 99.1, C-21) and *α, β*-unsaturated lactone (*δ_C_* 169.0, C-20; 120.4, C-22 and 170.9, C-23) signals, aligning with the spectroscopic data for γ-hydroxybutyrolactone units observed in related structures [41] (Figure 1). HMBC interactions between H_2_-16 and C-20; H-17 to C-20, C-21, and C-22; H-21 to C-22 and C-23; and H-22 to C-21 and C-23 supported the identification of a hydroxybutyrolactone structure linked to C-17, akin to that found in munronolide [40]. The NMR data indicate that the distinction between compound **3** and munronolide is primarily in the C-12 AcO and C-3 cinnamoyl groups. The AcO group is linked to C-12 via HMBC correlations from H-12 (*δ*_H_ 4.87, m) to the AcO moiety (*δ_C_* 170.9). The cinnamoyl group is attached to C-3 of compound **3** through an HMBC correlation from H-3 (*δ_H_* 4.18) to C-1′ (*δ_C_* 165.4) (Figure 2). The relative configuration of compound **3** was determined using ROESY experiments (Figure S1). The absolute configuration of 1*S*, 3*R*, 4*R*, 5*R*, 6*R*, 7*S*, 8*R*, 9*S*, 10*R*, 12*S*, 13*S*, and 17*R* for compound **3** was established by comparing experimental and computed ECD data (Figure S2). Based on this evidence, compound **3** was identified as a new malonolide derivative named 1,3-deacetyl-3-O-cinnamoyl munronolide 12-acetate, with cinnamoyl and AcO groups at C-3 and C-12, respectively.

Compound **4**, as shown by HRESIMS, has the same molecular formula as compound **3**, C_37_H_44_O_10_. The NMR spectra of compound **4** closely resemble those of compound **3**, except for the absence of the 21-hydroxybut-20(22)-en-21,23-γ-lactone ring signal found in compound **3**. Instead, signals indicating a 23-hydroxybut-20(22)-en-21,23-γ-lactone cycle at the C-17 position are observed, evidenced by ^1^H (*δ*_H_ 6.43(H-23) and 6.85(H-22) and ^13^C [*δ*_C_ 137.5(C-20), 171.4(C-21), 146.5(C-22), and 96.5(C-23)]) data [19] (Figure 1). The HMBC cross-correlation of H-17 (*δ*_H_ 2.94) to C-20 and C-21, and H-22 (*δ*_H_ 6.85) to C-17, C-20, C-21, and C-23, and the NOE correlation of the ^1^H- signal of H-7*β* (*δ*_H_ 4.22) with H-16*β* (*δ*_H_ 2.38), and H-16*β* with H-17*β* of **4** demonstrated that the γ-lactone ring is situated at C-17 and oriented to α. The AcO group is positioned at C-1 of compound **4**, as indicated by HMBC correlations from H-1 (*δ*_H_ 4.86) to the AcO group (*δ_C_* 170.8). The OH group is linked to C-12, confirmed by the C-12/12-OH cross-peak in the HMBC spectrum (Figure 2). The relative configurations of compound **4** were assessed from the NOESY data and compared with the NMR data for compounds **3** and **4** (Figure S1). The absolute configurations of (1*S*, 3*R*, 4*R*, 5*R*, 6*R*, 7*S*, 8*R*, 9*S*, 10*R*, 12*S*, 13*S*, and 17*R*) for compound **4** were determined by the similarity of the computed ECD spectra (Figure S2). Consequently, the structure of compound **4** was elucidated as 3-deacetyl-3-O-cinnamoyl-12-hydroxy-17 (4-hydroxy-2-buten-4-olide-2-yl) munronolideand named munronolide A.

Compound **5** was characterized as an amorphous powder with a molecular formula C_34_H_46_O_11_ evidenced by (HRESIMS) with an ionization of *m/z* 653.2935 [M + Na]^+^, calculated for C_34_H_46_NaO_11_ as 653.2932. This corresponds to a degree of unsaturation of 12. The ^1^H-NMR spectra displayed signals for four methyl groups (δ_H_ 1.79, 1.46, 1.12, 0.89), a methoxy group (δ_H_ 3.37), an acetyl group (δ_H_ 2.02), and a Tig group (δ_H_ 6.88, 1.78, and 1.73). The ^13^C-NMR spectra revealed three ester groups (δ_C_ 170.8, 170.7, and 166.7) and carbons associated with two double bonds (δ_C_ 146.1, 140.3, 137.5, and 128.6). A proton singlet at δ_H_ 5.90 and 5.82, along with carbon resonances at δ_C_ 170.8, 118.7, and 98.7, indicate a 21-hydroxybutenolide unit within the structure of **5**, as compared to meliazedalide A [42]. The presence of a methoxyl moiety at the C-12 position instead of a hydroxyl moiety, as in meliazedalide A (Figure 1), is corroborated by HMBC cross-peaks from H-OMe, H_2_-11, and H-9 to C-12, confirmed by a downfield chemical shift to δ_C_ 97.8 (Δδ_C_ +6.0 ppm) for C-12 (Figure 2). NOESY spectra of compound **5** and melazadine A reveal similar relative conformations (Figure S1). Based on computed ECD curves, the absolute configurations of compound **5** are proposed as (1*S*, 3*R*, 4*R*, 5*R*, 6*R*, 7*S*, 8*R*, 9*S*, 10*R*, 12*S*, 15*S*, and 17*R*) (Figure S2). Consequently, compound **5** was identified as 12-α-methylmeliazedalide A (Figure 1).

By comparing the measured ^1^H-NMR, ^13^C-NMR, and MS data with those reported in the literature, known limonoids were identified as 12*β*-hydroxynimbolinin A (**6**) [39], nimbolinin A (**7**) [39], nimbolinin D (**8**) [39], and nimbolinin C (**9**) [43] (Figure 1).

### 3.2. Effects of Isolate on Nitrite Levels in Macrophages

Previous studies have documented the use of *M. azedarach* fruit as a traditional remedy for various conditions. However, detailed pharmacological studies on its anti-inflammatory effects and mechanisms are limited. This study explored the inhibitory effects of the bioactive components isolated from *M. azedarach* on macrophage-mediated inflammation. Key inflammatory factors like TNF-α, IL-6, and NO either inhibit or promote inflammatory responses through multiple pathways. In this investigation, along with one new tirucallane triterpenoid (**1**), four new limonoids (**2**–**5**) and four known limonoids (**6**–**9**) were identified. These compounds were characterized as 21*β*-hydroxy bourjutinolone A (**1**), 3-acetyl-7-benzoylnimbidinin (**2**), 1,3-deacetyl-3-O-cinnamoyl munronolide 12-acetate (**3**), Munronolide A (**4**), and 12-α-methylmeliazedalide A (**5**), respectively.

The anti-inflammatory activities of constituents **1**–**9** were assessed by measuring nitrite levels in macrophages. Nitrites, which can either inhibit or promote inflammation, activate NF-κB; this transcription factor then spurs the liberation of pro-inflammatory cytokines like IL-6 and TNF-α, thereby accelerating the inflammatory process [44]. Inhibition of nitrite synthesis may thus mitigate inflammation. In this study, the effects of a new tirucallane triterpenoid (**1**), four new limonoids (**2**–**5**), and four known limonoids (**6**–**9**) on nitrite levels in LPS-mediated macrophages were assessed using the Griess method (Table 3). Limonoids **1**, **2**, and **5** showed potent anti-inflammatory effects. Pre-treatment with these limonoids significantly reduced nitrite production induced by LPS, with limonoid **2** demonstrating the strongest inhibition, exhibiting an IC_50_ value of 22.04 μM. However, the specific anti-inflammatory mechanism of limonoid **2** requires further exploration. Additionally, cytotoxicity tests using the SRB assay revealed that limonoids **1**–**9** were non-toxic to macrophages at concentrations up to 40 μM.

### 3.3. Inhibition of the Inflammatory Response Through Limonoid ***2***

**Figure 3:**
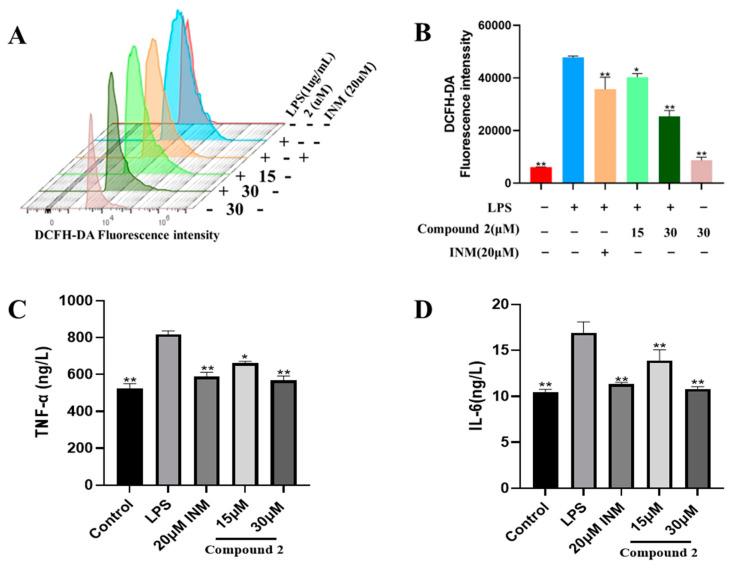
Compound **2** reduced the LPS-mediated pro-inflammatory response of RAW264.7 cells. (**A**) RAW264.7 cells were preconditioned by compound **2** (15, 30 μM) for 4 h and subsequently incubated with LPS for 24 h. Cells were marked by DCFH-DA for 20 min and measured by flow cytometry. (**B**) Statistical analysis of the fluorescence intensity of reactive oxygen species (ROS). (**C**,**D**) The cells were preconditioned by compound **2** (15, 30 μM) for 4 h and then incubated with LPS for 24 h. The supernatant was gathered, and the pro-inflammatory factors TNF-α and IL-6 were detected using an ELISA kit. * *p* < 0.05, ** *p* < 0.01, compared with the LPS group alone.

The expressions of inflammatory mediators like TNF-α and IL-6 are mainly regulated by NF-κB, which is a crucial component of the inflammatory process [9]. ROS also play an important role in inflammation, particularly in the LPS-mediated generation of the pro-inflammatory agents TNF-α and IL-6 and activation of the NF-κB cascade [45,46,47]. TNF-α and IL-6 are significant markers of inflammation in LPS-induced macrophage activation. ROS are crucial signaling molecules in the inflammatory process [45]. As depicted in Figure 3A,B, the flow cytometry results demonstrated a significant increase in ROS production in macrophages following LPS treatment. In contrast, limonoid **2** inhibited ROS production in a dose-dependent manner (Figure 3A,B). Furthermore, the levels of IL-6 and TNF-α were also elevated in macrophages [48,49] following LPS stimulation. However, limonoid **2** significantly decreased the levels of these cell factors in LPS-stimulated macrophages (Figure 3C,D), indicating its potential as an effective anti-inflammatory agent.

### 3.4. Exploring the Role of Limonoid ***2*** in the NF-κB Cascade

**Figure 4:**
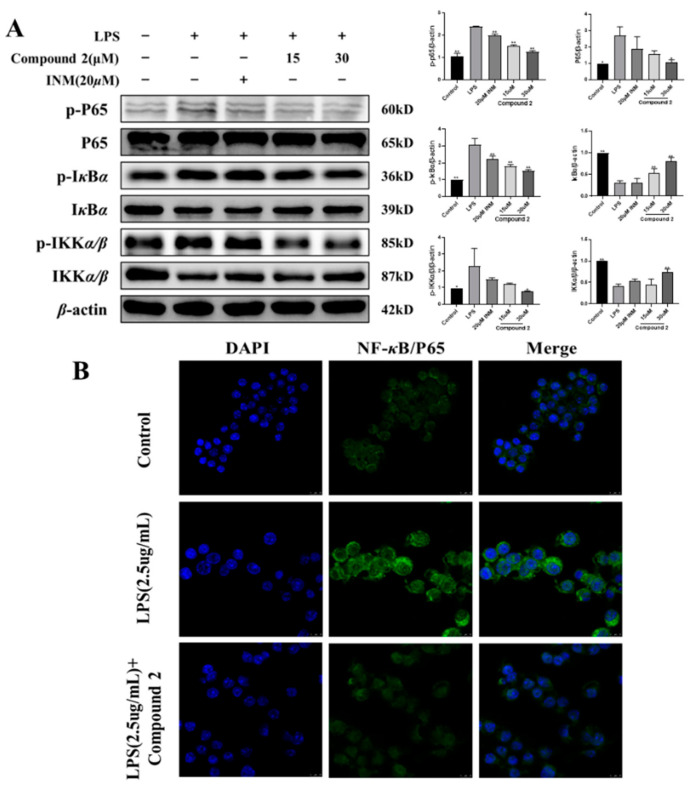
The NF-κB pathway is implicated in the anti-inflammatory process of limonoid **2**. (**A**) RAW264.7 cells were pre-treated by limonoid **2** (15, 30 μM) for 4 h and then mediated by LPS for 24 h. The expression of proteins p65, p-p65, IκBα, p-IκBα, IKKα/β, and p-IKKα/β was measured by Western blotting. (**B**) RAW264.7 cells were pre-treated by limonoid **2** for 4 h and then treated with LPS for 24 h. Translocation of p65 was detected by immunofluorescence, as outlined in the Methods sections. Primary anti-NF-κBp65 (1:500), secondary anti-fluorescein-coupled Goat Anti-Rabbit IgG (H + L) (1:100). * *p* < 0.05, ** *p* < 0.01, compared with the LPS group alone. The scale bar represents 10 μm.

Transcription factor-NF-κB plays a crucial role in LPS-mediated inflammatory procedures [47]. It is known to regulate various inflammatory mediators like IL-6, TNF-α, iNOS, and JAK2 [50]. The NF-κB signaling cascade is also pivotal in developing chronic infectious diseases. Studies have shown that in macrophages activated by LPS, NF-κB is sequestered in the cytoplasm until the upregulation of IKKα/β, which is crucial for NF-κB activation and promotes the phosphorylation and subsequent degradation of IκB [51]. It is probable that NF-κB p65 is phosphorylated on dephosphorylated IκB and translocated to the nucleus, thereby enhancing the release of pro-inflammatory factors and accelerating inflammatory damage [49]. We hypothesize that the NF-κB cascade may be implicated in the anti-inflammatory effects of limonoid **2**. Our current research indicates that the expressions of p65, p-IKKα, p-IKKβ, and p-IκBα/β in LPS-stimulated macrophages were elevated but were significantly reduced by limonoid **2** (Figure 4A). Additionally, immunofluorescence staining revealed that limonoid **2** inhibited the nuclear translocation of NF-κB p65 (Figure 4B), suggesting that the NF-κB cascade contributes to the anti-inflammatory action of limonoid **2**.

### 3.5. Exploring the Role of Limonoid ***2*** in iNOS and JAK2 Cascades

**Figure 5:**
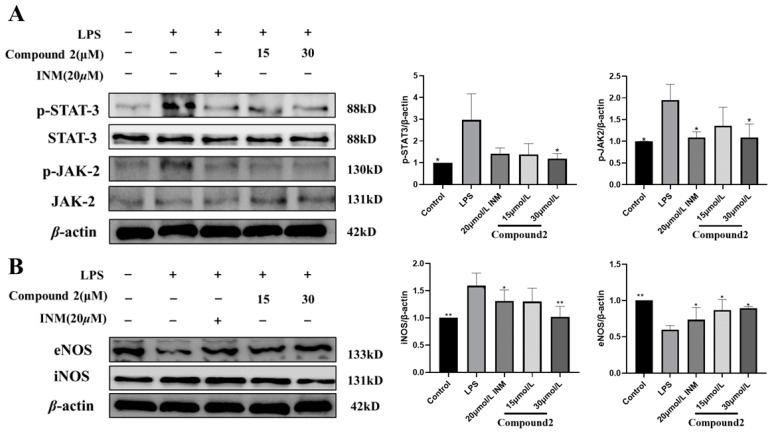
JAK2 and iNOS cascades are implicated in the anti-inflammatory process of limonoid **2**. (**A**) RAW264.7 cells were pre-treated by limonoid **2** (15, 30 μM) for 4 h and then treated with LPS for 24 h. Protein expression of p-STAT-3, STAT-3, p-JAK-2, and JAK-2 was measured by Western blotting. (**B**) RAW264.7 cells were pre-treated by limonoid **2** (15, 30 μM) for 4 h and then treated with LPS for 24 h. The expression of eNOS and iNOS proteins was detected by Western blotting. * *p* < 0.05, ** *p* < 0.01, compared with the LPS group alone.

To determine the impact of limonoid **2** on the activation of JAK2 and STAT3, we analyzed the level of p-JAK2 and p-STAT3 in both cytoplasmic and nuclear extracts via Western blotting. Results depicted in Figure 5A show that limonoid **2** partially inhibited the translocation of p-JAK2 and p-STAT3, indicating that limonoid **2** reduces LPS-induced JAK2 activity by blocking p-JAK2/STAT3 in the cytoplasm. Consequently, the anti-inflammatory properties of limonoid **2** may stem from its modulation of the JAK2/STAT3 signaling pathway. In this study, limonoid **2** preconditioning decreased iNOS levels and increased eNOS protein expression in a dose-dependent manner in LPS-mediated macrophages (Figure 5B). These findings suggest that limonoid **2** exhibits anti-inflammatory and antioxidant effects in LPS-mediated macrophages by suppressing the induction of JAK2 and eNOS.

## 4. Discussions

Limonoids, derived from triterpenes, are unique, structurally complex, and highly oxidized phytochemicals known for their extensive rearrangements. Inflammation, the body’s natural response to infections and chronic diseases, can escalate into various health complications, including cancer. The anti-inflammatory activities of limonoid compounds have, therefore, been targeted to identify potential therapeutic agents for inflammation management. Beyond their structural diversity, the biological activities of limonoids have garnered significant attention due to their broad spectrum of biological properties, including insecticidal, antimicrobial, antimycotic, antimalarial, cytotoxic, antiviral, and anti-inflammatory effects. Research into anti-inflammatory limonins is crucial for developing new chemical entities to treat inflammatory and immune disorders.

In this study, a new tirucallane triterpenoid (**1**), four novel limonoids (**2**–**5**), and four known limonoids (**6**–**9**) were separated from the fruits of *M. azedarach*. The structural analysis of isolated compounds was primarily conducted using HRMS and NMR data. Stereochemical localizations were mainly determined through rule-of-thumb-based ^1^H NMR and ^1^H NMR homonuclear decoupling experiments. Among the isolates, tirucallane triterpenoid (**1**) and limonoids **2** and **5** exhibited potent anti-inflammatory activities against LPS-induced macrophages. Notably, limonoid **2** demonstrated superior inhibition of nitrite production with an IC_50_ value of 22.04 μM. The anti-inflammatory and antioxidative mechanisms of limonoid **2** were further investigated, revealing that this compound modulates the NF-κB and JAK2 signaling pathways.

Free radicals, like ROS and reactive nitrogen species (RNS), are unstable molecules that lack an electron and seek stability by accepting electrons or hydrogen atoms. Certain compounds, known for their high antioxidant activity, can donate electrons or hydrogen, thereby stabilizing various free radicals and ROS effectively due to their resonance stability [52]. In this context, various limonoids exhibit remarkable antioxidant activity by supplying electrons or hydrogen atoms to stabilize free radicals [53]. In the current study, limonoid **2** inhibited ROS generation in a dose-dependent manner in LPS-mediated RAW264.7 cells, suggesting that limonoid **2** possesses both anti-inflammatory and antioxidant properties through the modulation of ROS generation.

Macrophages, integral components of the immune system, initiate immune responses through the secretion of NO and factors in reaction to outside stimuli or by phagocytosis to eliminate foreign substances. Prolonged reactions may lead to chronic inflammation, contributing to various diseases [54]. In this study, limonoid **2** demonstrated significant antioxidant activity and played a crucial anti-inflammatory role by inhibiting the generation of NO, inflammatory factors, and ROS.

NO is synthesized by nitric oxide synthase (NOS). Inducible NOS (iNOS) is vital in the inflammatory response [55]. NO produced intracellularly can react with ROS to form RNS, which induce oxidative injury to macromolecules and accelerate inflammation-mediated cellular injury by impeding mitochondrial function [56,57]. Previous research has shown that materials with strong antioxidant activity can utilize ROS to suppress NO production [58]. This study shows that limonoid 2, due to its potent antioxidant capabilities, may inhibit NO production via an LPS-mediated reduction in ROS generation.

Activated macrophages secrete TNF-α, IL-6, and IL-1β and are typical inflammatory cytokines. They can activate other immune cells or enhance macrophage activation through autocrine effects [59]. Overproduction of these cytokines can promote apoptosis and lead to tissue injury [60]. Limonoid **2** reduced the production of these cytokines in LPS-stimulated macrophages, exhibiting anti-inflammatory activity.

The nuclear transcription factor NF-κB is recognized for its pivotal role in modulating the expressions of different pro-inflammatory markers [61]. Thus, inhibiting NF-κB activation is central to the pharmacological mechanism of anti-inflammatory agents [62]. In response to inflammatory stresses like LPS, NF-κB activation leads to IκB-α phosphorylation and degradation and nuclear translocation of NF-κB p65 protein. Translocated p65 interacts with NF-κB bonding sites to activate the transcription of anti-inflammatory agents [63]. We demonstrated that treatment with limonoid **2** suppresses both the phosphorylation of IκB-α and the nuclear translocation of NF-κB p65 proteins.

Pro-inflammatory factors, like iNOS, eNOS, JAK2, and STAT3, play crucial roles in various inflammation models due to their regulatory effects [64,65,66]. Studies have shown that in these models, the overproduction of NO, which enhances iNOS expression, can subsequently influence the expression of JAK2. Consequently, iNOS and JAK2 are identified as potential targets for inflammation mitigation [55]. In this study, limonoid **2** was observed to diminish LPS-mediated JAK2 activity by inhibiting the phosphorylation of JAK2/STAT3 in the cytoplasm. Preconditioning with limonoid **2** reduced iNOS and increased eNOS protein expression in macrophages. These outcomes demonstrate that limonoid **2** inhibits JAK2 phosphorylation, thereby preventing macrophage activation in response to extracellular stimuli. These results indicate that the eNOS and NF-κB pathways might underpin the anti-inflammatory activities of limonoid **2**.

This study had limitations. First, the effects of limonoid 2 observed after 4 h may not reflect its effects at different points. In future research, we can compare the differences in therapeutic effects of limonoid **2** with different durations of its action. Second, the impact of limonoid **2** on ROS production has not been investigated, so in vivo animal studies are also required to validate the mechanism of action further. Consequently, future studies should address these limitations to better understand its antioxidant and anti-inflammatory effects.

## 5. Conclusions

This study examined the active components and anti-inflammatory mechanisms of *M*. *azedarach* from chemical and pharmacological perspectives, identifying nine constituents from *M. azedarach* fruits, including one novel tirucallane triterpenoid (**1**), four new limonoids (**2**–**5**), and four known limonoids (**6**–**9**). Limonoid **2** exhibited significant anti-inflammatory effects by reducing nitrite levels, modulating ROS synthesis, and decreasing IL-6 and TNF-α. The eNOS and NF-κB pathways are integral to the anti-inflammatory effects of limonoid **2**. Collectively, these findings suggested that limonoid **2** considerably diminishes inflammation. Future in vivo studies are needed to confirm these properties of limonoid **2** and elucidate its potential impact on combating inflammation and oxidative stress.

## Figures and Tables

**Table 1:** ^1^H NMR (600 MHz) data of compounds **1**–**5** (CDCl_3_).

Proton	1	2	3	4	5
1	1.49(m)	3.59(t,2.8)	3.86(t,3.9)	4.86(m)	3.56(s)
	2.01(m)				
2	2.26(dt,11.2,3.2)	2.02(dd,2.8,15.1)	1.09(dd,2.0,15.1)	1.06(dd,1.5,10.9)	2.15(m)
	2.78(dt,14.7,3.8)	2.34(dd,3.2,15.1)	2.28(dd,2.3,15.1)	2.38(dd,2.8,10.9)	2.19(m)
3		5.08(t,2.7)	4.94(t,4.1)	4.89(t,4.1)	4.86(t,2.5)
5	1.75(m)	2.67(d,12.6)	2.45(d,12.1)	2.66(d,4.8)	2.76(d,12.7)
6	2.11(m)	4.28(d,12.6)	4.17(d,12.1)	4.16(d,12.2)	4.01(dd,12.5,3.0)
7	5.34(t,3.1)	5.90(d,3.0)	4.19(d,2.8)	4.22(d,3.3)	5.68(d,2.8)
9	2.32(m)	3.58(dd,5.5,7.3)	2.64(dd,5.5,6.9)	2.94(dd,3.6,4.4)	2.98(d,7.5)
11	1.61(m)	2.54(m)	2.28(m)	2.28(m)	1.72(m)
		2.34(m)	2.03(m)	2.08(m)	1.78(m)
12	1.60(m)		4.87(m)	3.85(m)	5.94(s)
	2.03(m)				
15	1.62(m)	5.73(t,2.5)	5.66(t,3.7)	5.64(t,3.5)	4.95(7.4)
16	1.51(m)	2.34(m)	2.45(m)	2.38(m)	1.77(m)
	1.91(m)	2.43(m)	2.64(m)	2.51(m)	2.19(m)
17	1.85(m)	3.46(m)	2.90(m)	2.94(m)	3.36(m)
18	0.84(s)	0.97(s)	0.95(s)	0.91(s)	1.79(s)
19	1.03(s)	0.98(s)	1.00(s)	1.03(s)	0.89(s)
20	2.03(m)				
21	5.48(d,2.2)	7.25(t,1.6)	5.88(s)		6.09(s)
22	2.02(m)	6.48(d,1.6)	5.85(s)	6.85(d,1.2)	5.82(m)
	1.12(m)				
23	4.66(d,5.1)	7.27(s)		6.43(d,1.8)	
24	3.37(d,5.2)				
26	1.18(s)				
27	1.17(s)				
28	1.07(s)	3.18(d,7.8)	3.62(d,7.6)	3.62(d,7.6)	3.47(d,7.6)
		3.46(d,7.8)	4.12(d,7.6)	4.11(d,8.0)	3.56(d,7.6)
29	1.14(s)	1.17(s)	1.15(s)	1.15(s)	1.12(s)
30	1.06(s)	1.18(s)	1.09(s)	1.09(s)	1.46(s)
12-OCH3					3.37(s)
1-OAC				1.83(s)	
3-OAC		1.82(s)			2.02(s)
12-OAC			2.03(s)		
2’			6.40(d,15.9)	6.03(d,17.9)	
3’		8.05(dd,7.6,1.3)	7.72(d,16.0)	7.71(d,15.9)	6.88(qd,7.0,1.5)
4’		7.39(t,7.6)			1.73(d,7.0)
5’		7.53(t,7.5)	7.48(dd,7.2,1.7)	7.49(dd,4.4,1.9)	1.78(s)
6’		7.39(t,7.6)	7.38(t,4.9)	7.39(t,6.9)	
7’		8.05(dd, 7.6,1.3)	7.40(t,4.1)	7.40(t,8.1)	
8’			7.38(t,4.9)	7.39(t,6.9)	
9’			7.48(dd,7.2,1.7)	7.49(dd,4.4,1.9)	

**Table 2:** ^13^C NMR (150 MHz) data of compounds **1**–**5** (CDCl_3_).

Carbon	1	2	3	4	5
1	38.6	71.8	71.1	77.2	70.6
2	35.1	30.3	25.1	24.5	29.1
3	217.0	73.6	73.1	72.4	72.2
4	47.7	42.5	43.9	43.9	42.4
5	52.5	40.2	38.7	38.7	39.0
6	24.5	72.9	73.7	73.9	72.5
7	118.2	74.7	74.1	74.0	74.6
8	145.7	44.5	39.7	39.8	45.4
9	48.0	36.6	34.3	34.8	36.4
10	35.2	40.3	45.2	45.2	41.2
11	17.9	34.1	30.5	30.4	30.5
12	31.6	213.8	77.9	71.2	97.8
13	43.6	61.5	52.5	51.7	140.3
14	50.9	154.1	156.1	157.1	146.1
15	34.4	123.8	122.5	122.2	77.9
16	27.6	34.2	35.5	35.8	35.8
17	48.5	42.9	51.2	48.9	49.3
18	23.3	19.4	15.5	15.1	16.5
19	12.8	15.6	16.1	15.5	16.7
20	47.1	124.6	169.1	137.5	_ ^a^
21	101.8	142.4	99.1	171.4	98.7
22	33.6	112.6	120.4	146.5	118.7
23	76.7	141.0	170.9	96.5	170.8
24	86.5				
25	71.1				
26	25.9				
27	25.7				
28	24.6	78.1	78.4	78.4	78.1
29	21.7	18.8	20.2	20.2	19.0
30	27.4	25.8	26.7	26.9	20.7
12-OCH3					55.2
1-OAC				170.8	
				21.2	
3-OAC		169.2			170.7
		20.9			21.3
12-OAC			170.9		
			21.5		
1’		165.1	165.4	165.4	166.7
2’		130.5	117.0	117.2	128.6
3’		129.6	146.8	144.8	137.5
4’		128.6	133.8	134.1	14.6
5’		133.2	128.4	128.5	12.2
6’		128.6	129.2	129.2	
7’		129.6	131.	130.9	
8’			129.2	129.2	
9’			128.4	128.5	

^a^ Signal not clearly observable from 1D and 2D NMR.

**Table 3:** Anti-inflammatory activity for compounds **1**–**9** in vitro.

Compounds	IC_50_ (μM)	Compounds	IC_50_ (μM)
**1**	26.85 ± 1.56	**6**	64.95 ± 3.09
**2**	22.04 ± 0.96	**7**	64.95 ± 3.09
**3**	>100	**8**	50.80 ± 4.06
**4**	>100	**9**	>100
**5**	24.06 ± 1.92	Indomethacin ^a^	37.06 ± 2.56

^a^ Positive control.

## Data Availability

Data are included in the article.

## Supplementary Materials

The following supplementary data can be downloaded at https://www.mdpi.com/article/10.3390/antiox13111338/s1. Figures S1–S37: ROESY correlations, ECD, NMR, and HRMS spectra of new compounds **1**–**5**. Table S1. Information about the chemical reagents used in the experiment. Supplementary S1. Detailed procedures for Western blotting. Supplementary S2. Detailed procedures for Immunofluorescence.

## Abbreviations

Interleukin-6 (IL-6); Tumor Necrosis Factor-α (TNF-α); Nitric Oxide (NO); Inducible Nitric Oxide Synthase (iNOS); Janus Kinases 2 (JAK2); Nuclear Factor Kappa-B (NF-κB); Endothelial Nitric Oxide Synthase (eNOS); NF-kappa-B Inhibitor Alpha (IKB-α); Inhibitor of Kappa B KinaseAlpha/Beta (IKKα/β); Fetal Bovine Serum (FBS); Sulforhodamine B (SRB); Lipopolysaccharide (LPS).
