# Supplementary material for: Chemical Constituents from the Fruit of Melia azedarach and Their Anti-Inflammatory Activity
Source: Antioxidants (Basel). 2024 Oct 31;13(11):1338. doi: 10.3390/antiox13111338 (PMC11591037; doi:10.3390/antiox13111338)
Supplement: Supplementary file 1 [file antioxidants-13-01338-s001.zip › Supplementary File.pdf]

Chemical Constituents from the Fruit of *Melia azedarach* and Their Anti-Inflammatory Activity

List of Supporting Information

Supplementary S1. Detailed procedures for Western blotting.

Supplementary S2. Detailed procedures for Immunofluorescence.

Table S1. Information about the chemical reagents used in the experiment.

Figure S1. Key ROESY correlations of compounds **1–5**.

Figure S2 Calculated and experimental ECD spectra of compounds **1-5**.

Figure S3. ^{1}H NMR spectrum (600 MHz) of compound **1** in $CDCl_3$.

Figure S4. ^{13}C NMR spectrum (150 MHz) of compound **1** in $CDCl_3$.

Figure S5. HMQC spectrum of compound **1** in $CDCl_3$.

Figure S6. HMBC spectrum of compound **1** in $CDCl_3$.

Figure S7. ^{1}H-^{1}H COSY spectrum of compound **1** in $CDCl_3$.

Figure S8. NOESY spectrum of Compound **1** in $CDCl_3$

Figure S9. HRESIMS spectrum of compound **1**.

Figure S10. ^{1}H NMR spectrum (600 MHz) of compound **2** in $CDCl_3$.

Figure S11. ^{13}C NMR spectrum (150 MHz) of compound **2** in $CDCl_3$.

Figure S12. HMQC spectrum of compound **2** in $CDCl_3$.

Figure S13. HMBC spectrum of compound **2** in $CDCl_3$.

Figure S14. ^{1}H-^{1}H COSY spectrum of compound **2** in $CDCl_3$.

Figure S15. NOESY spectrum of Compound **2** in $CDCl_3$

Figure S16. HRESIMS spectrum of compound **2**.

Figure S17. ^{1}H NMR spectrum (600 MHz) of compound **3** in $CDCl_3$.

Figure S18. ^{13}C NMR spectrum (150 MHz) of compound **3** in $CDCl_3$.

Figure S19. HMQC spectrum of compound **3** in $CDCl_3$.

Figure S20. HMBC spectrum of compound **3** in $CDCl_3$.

Figure S21. ^{1}H-^{1}H COSY spectrum of compound **3** in $CDCl_3$.

Figure S22. NOESY spectrum of Compound **3** in $CDCl_3$

Figure S23. HRESIMS spectrum of compound **3**.

Figure S24. ^{1}H NMR spectrum (600 MHz) of compound **4** in $CDCl_3$.

Figure S25. ^{13}C NMR spectrum (150 MHz) of compound **4** in $CDCl_3$.

Figure S26. HMQC spectrum of compound **4** in $CDCl_3$.

Figure S27. HMBC spectrum of compound **4** in $CDCl_3$.

Figure S28. ^{1}H-^{1}H COSY spectrum of compound **4** in $CDCl_3$.

Figure S29. NOESY spectrum of Compound **4** in $CDCl_3$

Figure S30. HRESIMS spectrum of compound **4**.

Figure S31. ^{1}H NMR spectrum (600 MHz) of compound **5** in $CDCl_3$.

Figure S32. ^{13}C NMR spectrum (150 MHz) of compound **5** in $CDCl_3$.

Figure S33. HMQC spectrum of compound **5** in $CDCl_3$.

Figure S34. HMBC spectrum of compound **5** in $CDCl_3$.

Figure S35. ^{1}H-^{1}H COSY spectrum of compound **5** in $CDCl_3$.

Figure S36. NOESY spectrum of Compound **5** in $CDCl_3$

Figure S37. HRESIMS spectrum of compound **5**.

Suppl 1. Detailed procedures for Western blotting

The configuration should be carried out with an 8% rapid gel-making kit, with the sample quantity increased in accordance with the test results of BCA. The electrophoresis process should be conducted with rapid electrophoresis solution 225V for 30 minutes. Thereafter, the electrophoresed gel and PVDF membrane should be placed into the transfer device, followed by electrophoresis with ice-bath free rapid electrophoresis solution 400mA. The procedure should be continued for 30-40 minutes (according to the molecular weight), followed by a TBST rinse for five minutes. The strip should then be placed in a rapid closure solution shaker and closed for 30 minutes (or skimmed milk powder for two hours). TBST rinse three times for five minutes each time, and then proceed to cut the strip according to the target protein. Subsequently, the specimen is to be incubated in the refrigerator at 4 degrees Celsius for a period of 14 to 18 hours, protected from light, with the primary antibody solution. Following this, the specimen is to be rinsed three times for a period of 5 minutes each time with TBST. This is to be followed by incubation in the secondary antibody solution at room temperature for 1 hour, again protected from light. The specimen is then to be rinsed three times for a period of 5 minutes each time with TBST. Finally, the specimen is to be developed.

Suppl 2. Detailed procedures for Immunofluorescence

The cells were cultured in confocal Petri dishes. At the conclusion of the drug administration period, the cells were washed twice with PBS. They were then fixed with cold anhydrous ethanol for 30 minutes and subsequently rinsed three times with PBS for five minutes each time. 0.5% Triton X-100 was added to the cells at room temperature for 20 minutes to facilitate permeabilisation, after which they were rinsed three times with PBS for 5 minutes each time. The cells were then blocked with 1% BSA (prepared with PBS) for 30 minutes at room temperature. The primary antibody was subsequently added to the cells at 4 degrees Celsius. The samples should be refrigerated overnight (primary antibody diluted with 1% BSA, 1:500). The next day, the samples should be rinsed three times with PBST for 5 minutes each time. The fluorescent secondary antibody should then be incubated at room temperature and in the dark for one hour. The samples should be rinsed three times. Subsequently, the cells were incubated in a Petri dish with a drop of DAPI for 5 minutes in the dark, then washed three times with PBST. Finally, a few drops of anti-fluorescence quenching sealer were added, and the cells were observed with a confocal microscope.

Table S1. Information about the chemical reagents used in the experiment

Reagent	Brand
One-step PAGE colour gel ultra-rapid gel preparation kit (8%)	Servicebio
SWE Rapid High Resolution Electrophoresis Buffer	Servicebio
Ice bath free rapid membrane transfer buffer	Servicebio
Protein Free Rapid Sealing Solution	Servicebio
HyperSignal High Sensitivity ECL Chemiluminescent Substrate	4A Biotech
DMEM High Sugar	Servicebio
Fetal Bovine Serum	Scitecher
Sulforhodamine B	Yuanye
Nonfat- Dried Milk	Dingguo Changsheng
Recombinant Anti-beta-actinantibody(Mouse mAb)	Servicebio
Anti-NF-Kb p65 Recombinant Rabbit Monoclonal	Huabio
Rb mAb to NF-Kb p65（phospho s529）	Abcam
Anti-IKBαRecombinant Rabbit Monoclonal	Huabio
RbmAbtoIKB alpha（phospho Y42）	Abcam
Anti-IKKα+βRecombinant Rabbit Monoclonal	Huabio
Phospho- IKKα/β-S176/180 Rabbit pAb	ABclonal
Anti-JAK-2Recombinant Rabbit Monoclonal	Huabio
Rb mAb to JAK2（phospho Y1007+Y1008）	Abcam
Anti-STAT-3Recombinant Rabbit Monoclonal	Huabio
Rb mAb to STAT3（phospho Y705）	Abcam
iNOS Rabbit PloyAb	Proteintech
eNOSRabbit PloyAb	Proteintech
Goat anti-Rabbit IgG-HPR antibody	Huabio

Goat anti-Mouse IgG-HPR antibody	Huabio
BCA kit	Servicebio
Reactive Oxygen Species Assay Kit	Beyotime
RIPA lysate	Dingguo Changsheng
Protease inhibitor mixtures	Dingguo Changsheng
Phosphorylated protease complex inhibitor	Dingguo Changsheng
5*SDS protein electrophoresis buffer	Dingguo Changsheng
DAPI	Genview
BSA	Dingguo Changsheng
Triton X-100	Dingguo Changsheng
Fluorescein-conjugated Goat Anti-Rabbit IgG(H+L)	Dingguo Changsheng
Anti-fluorescence quenching encapsulant	Dingguo Changsheng
Mouse TNF-α ELISA Kit	MeiMian
Mouse IL-6 ELISA Kit	MeiMian

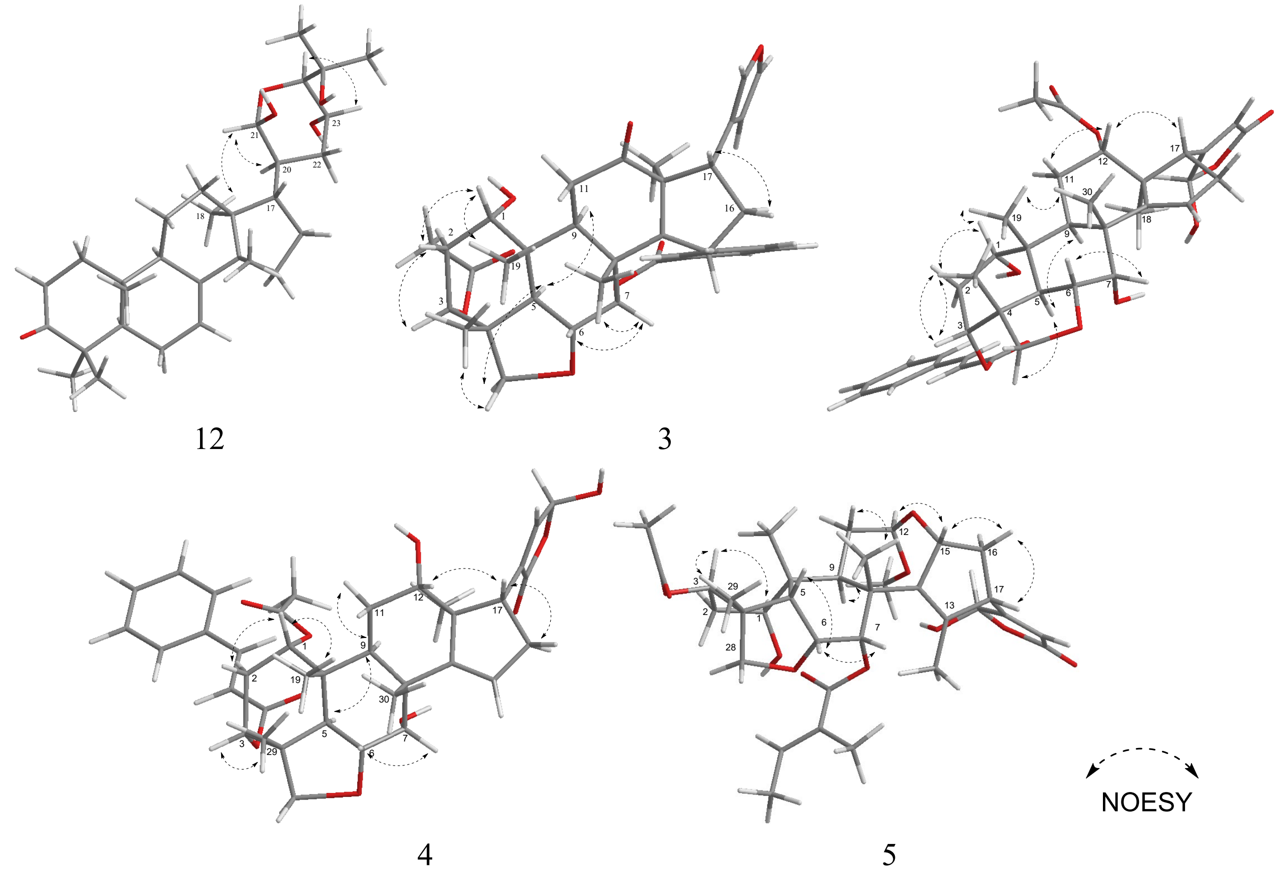

Figure **S1**. Key ROESY correlations of compounds **1–5**.

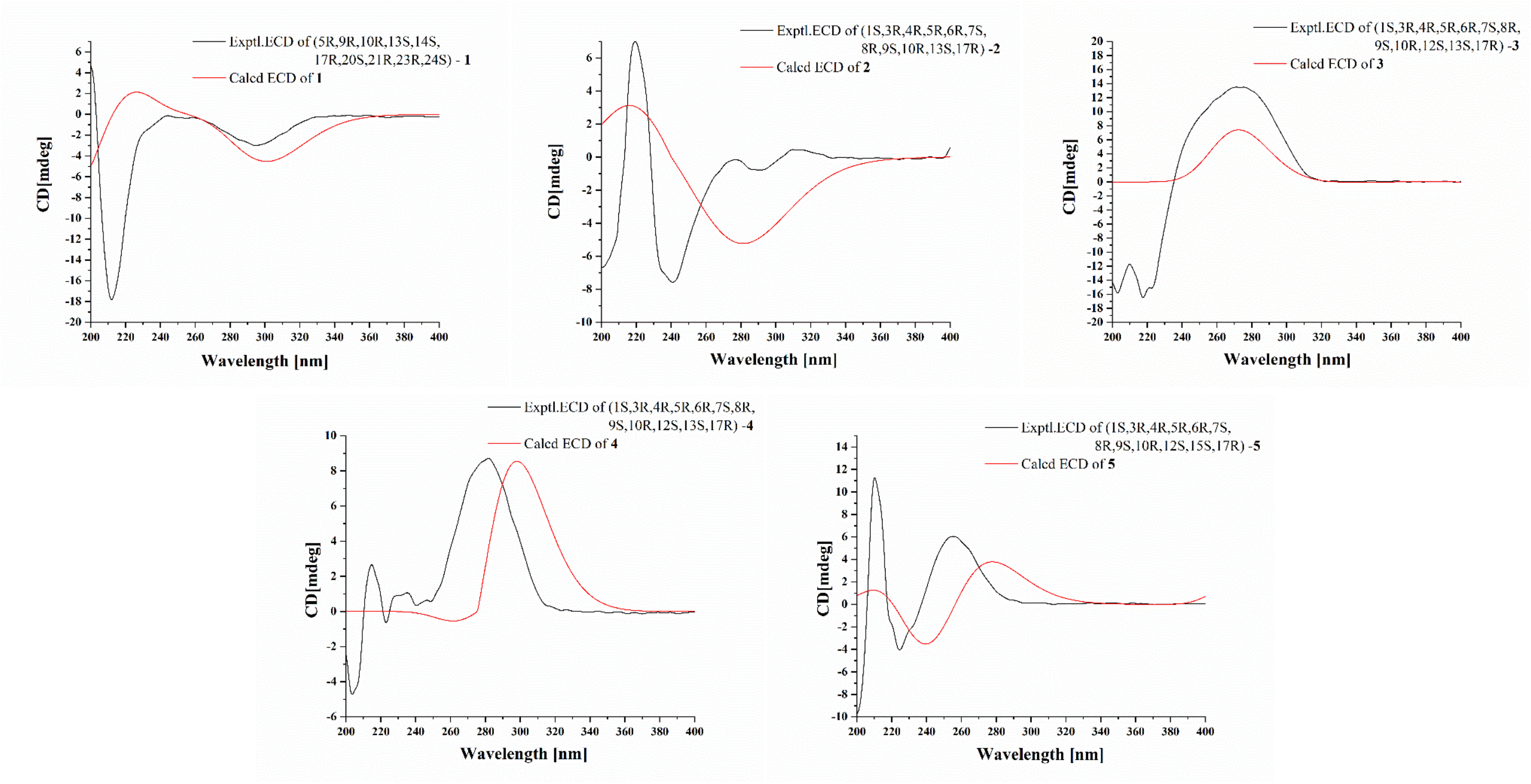

Figure S2 Calculated and experimental ECD spectra ofcompounds**1-5**.

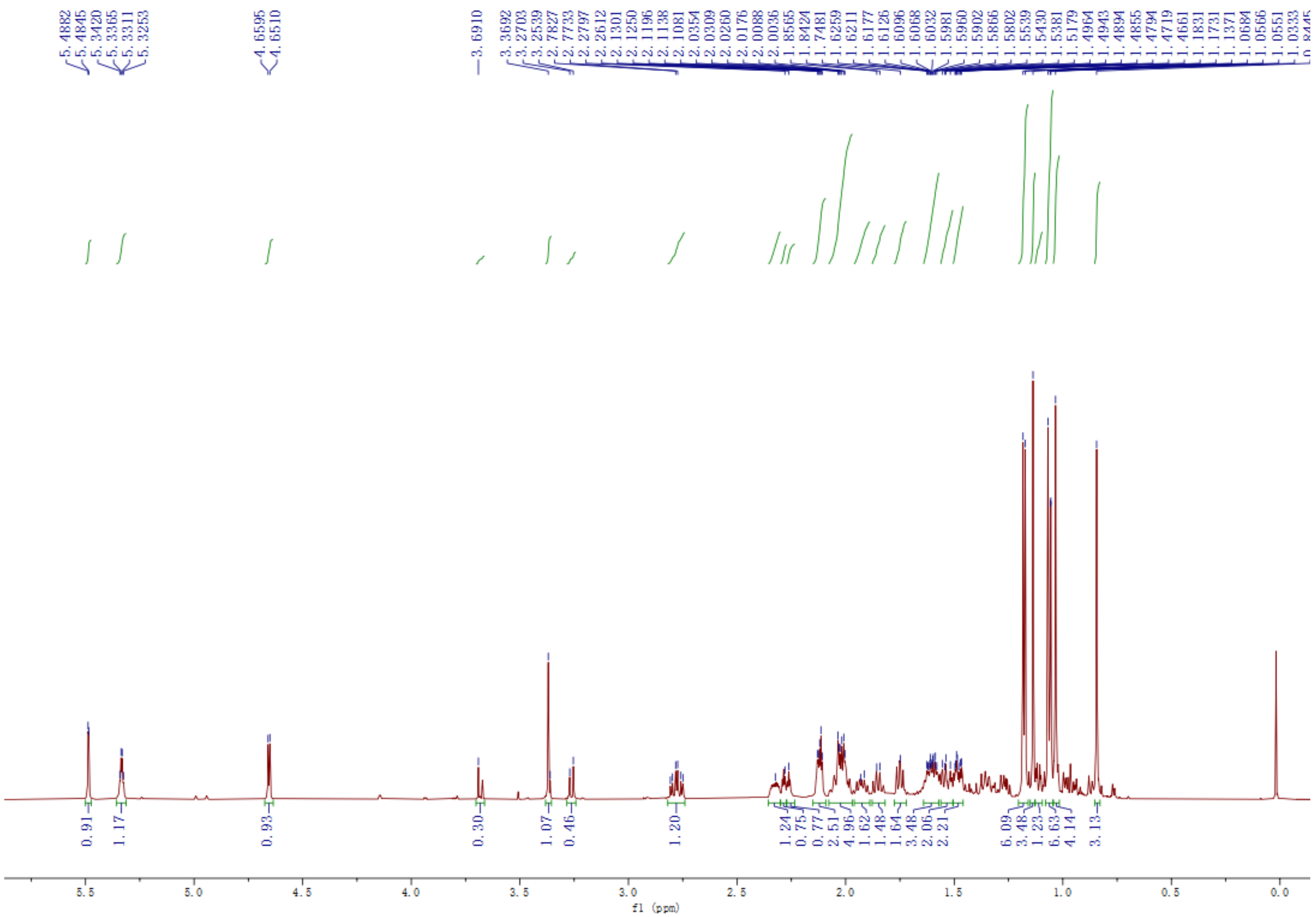

Figure S3. ^{1}H NMR spectrum (600 MHz) of compound **1** in $CDCl_3$.

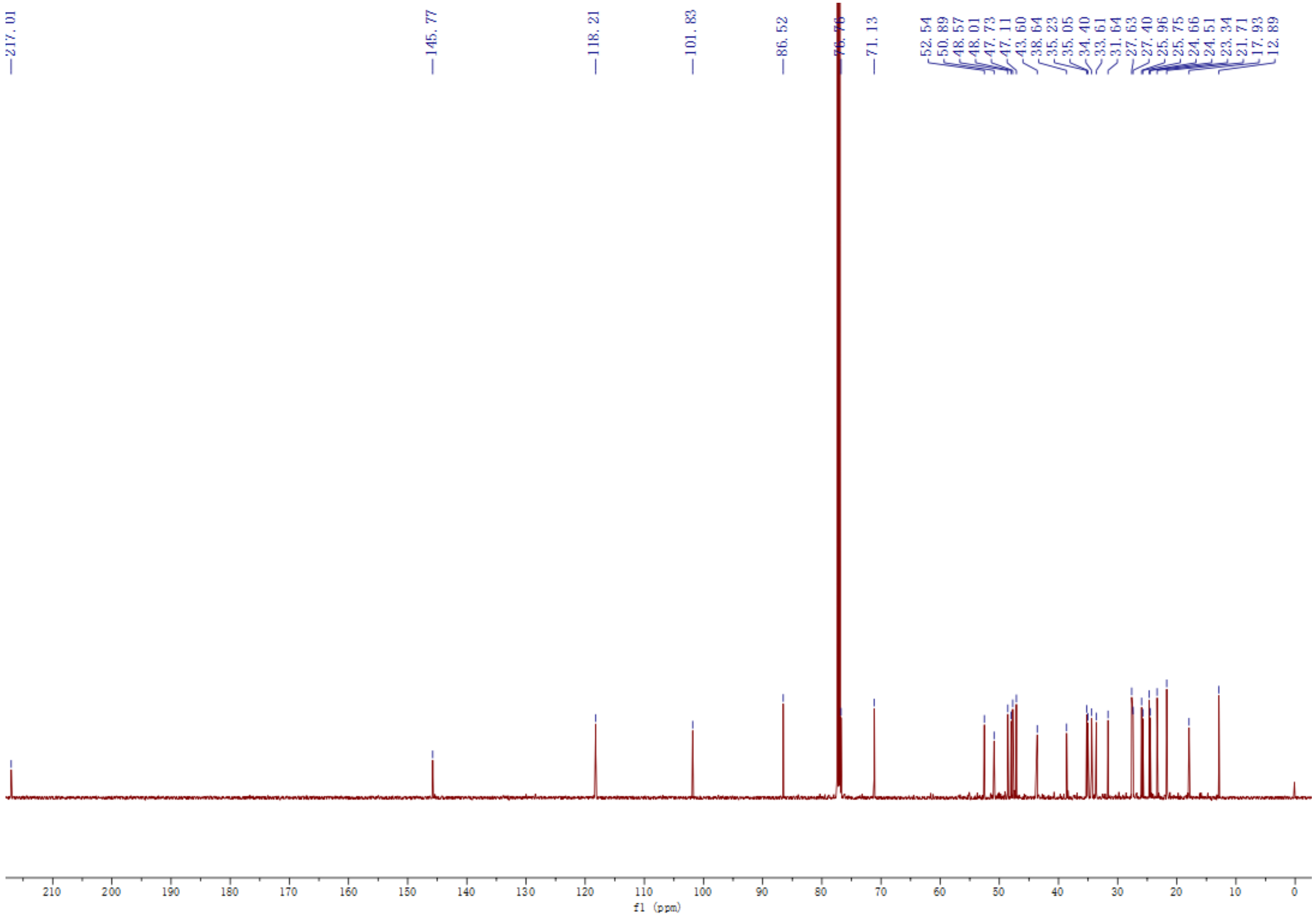

Figure S4. ^{13}C NMR spectrum (150 MHz) of compound **1** in $CDCl_3$.

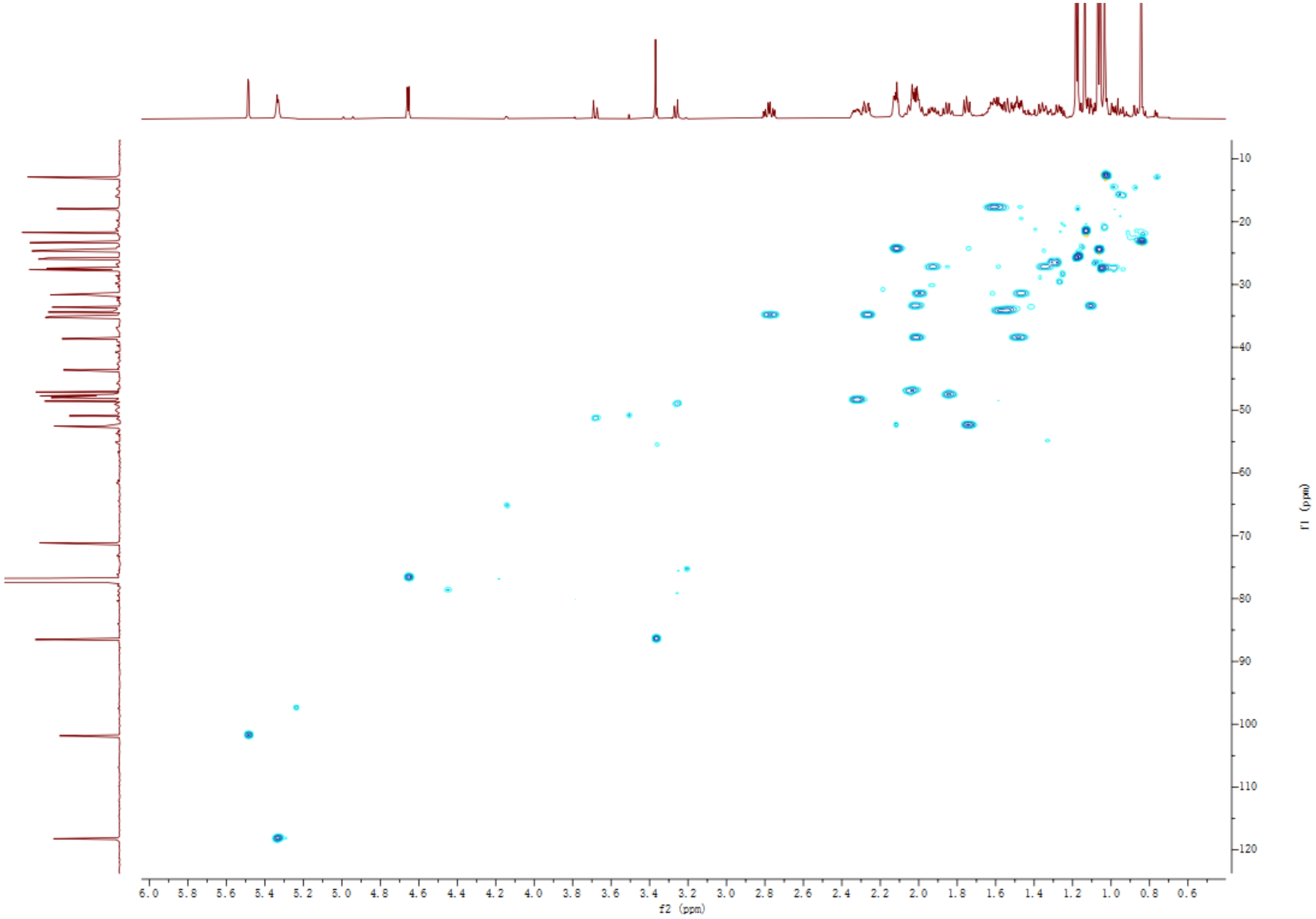

Figure S5. HSQC spectrum of compound **1** in $CDCl_3$.

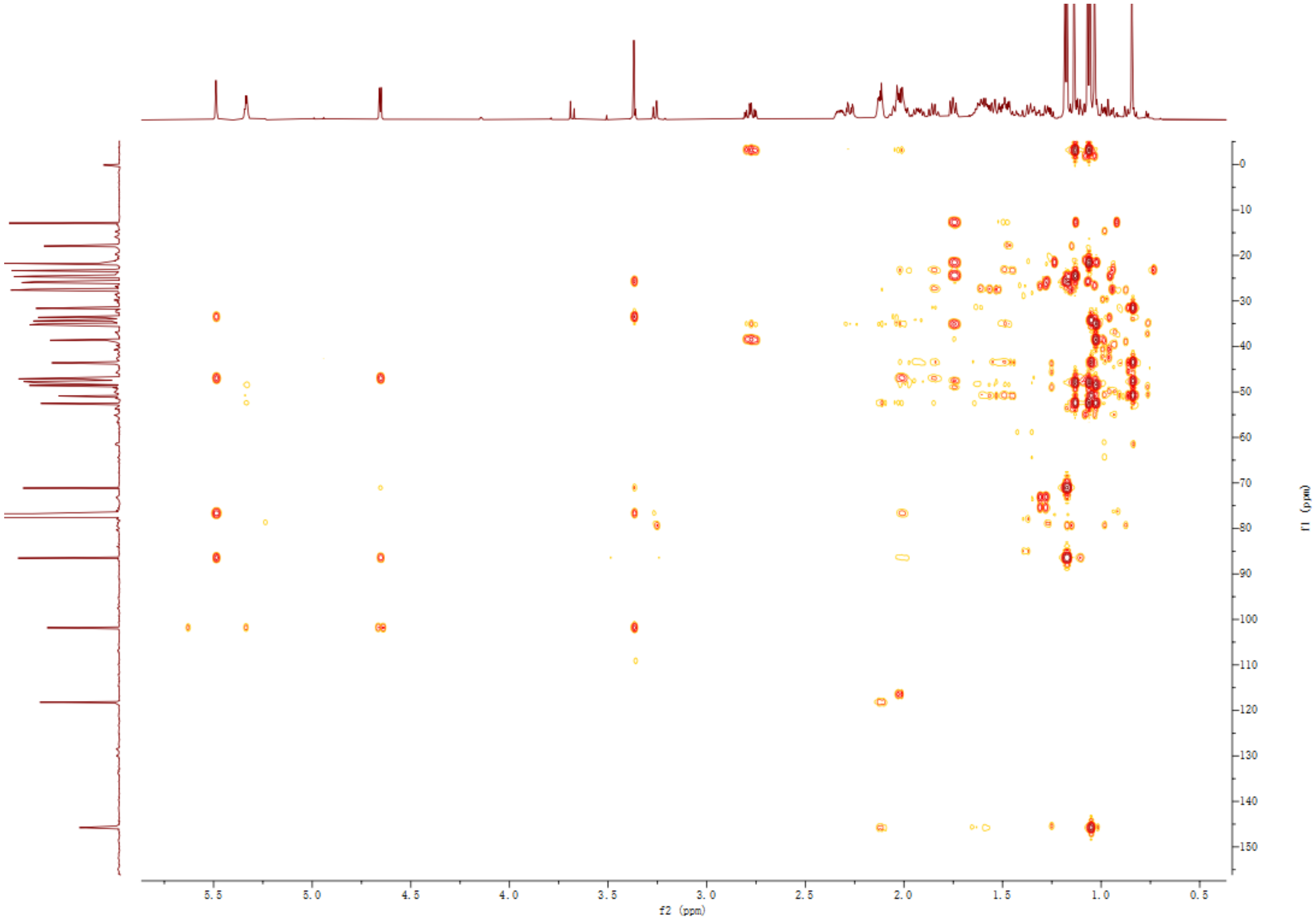

Figure S6. HMBC spectrum of compound **1** in $CDCl_3$.

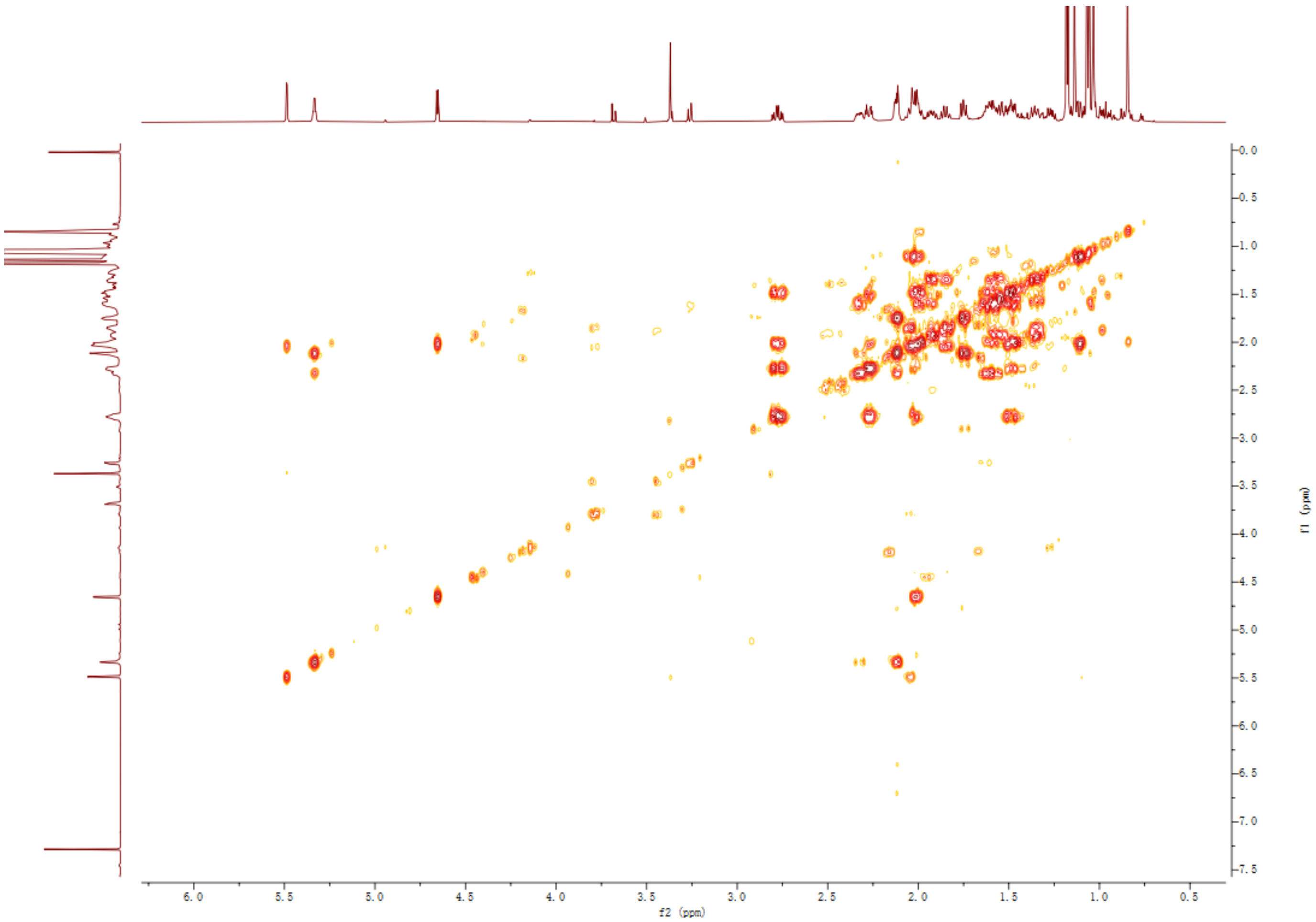

Figure S7. ^{1}H-^{1}H COSY spectrum of compound **1** in $CDCl_3$.

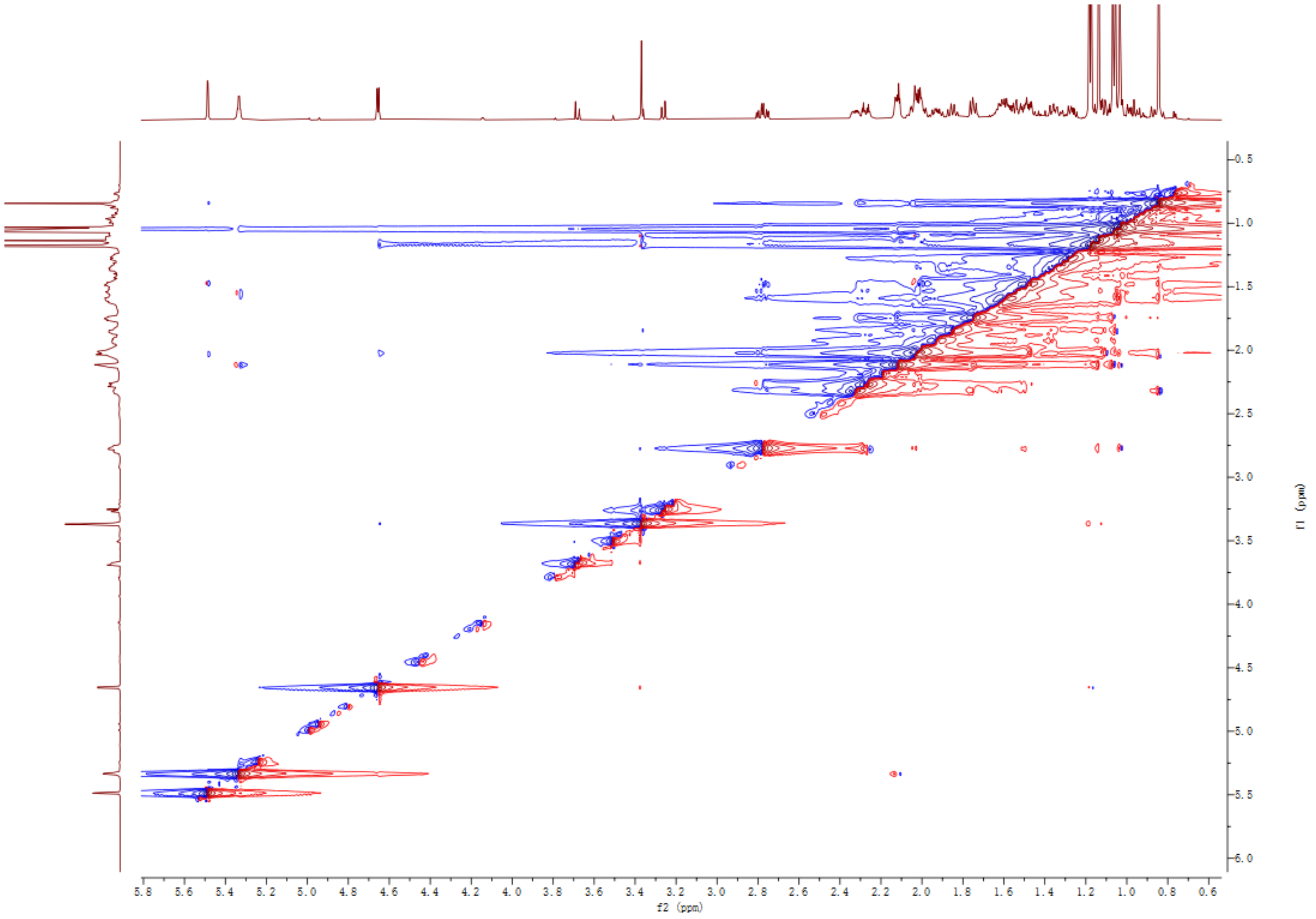

Figure S8. NOESY spectrum of Compound **1** in $CDCl_3$

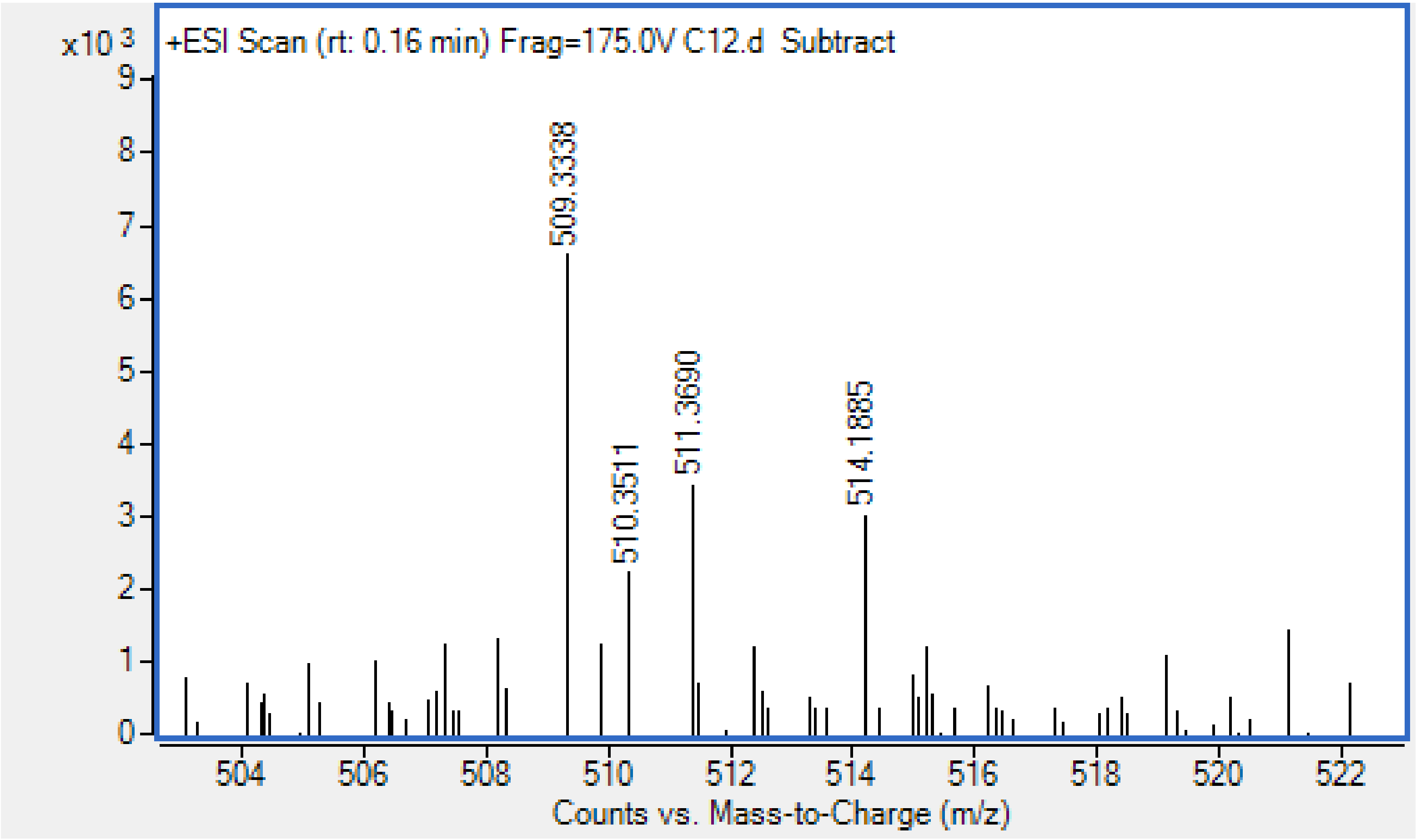

Figure S9. HRESIMS spectrum of compound **1**.

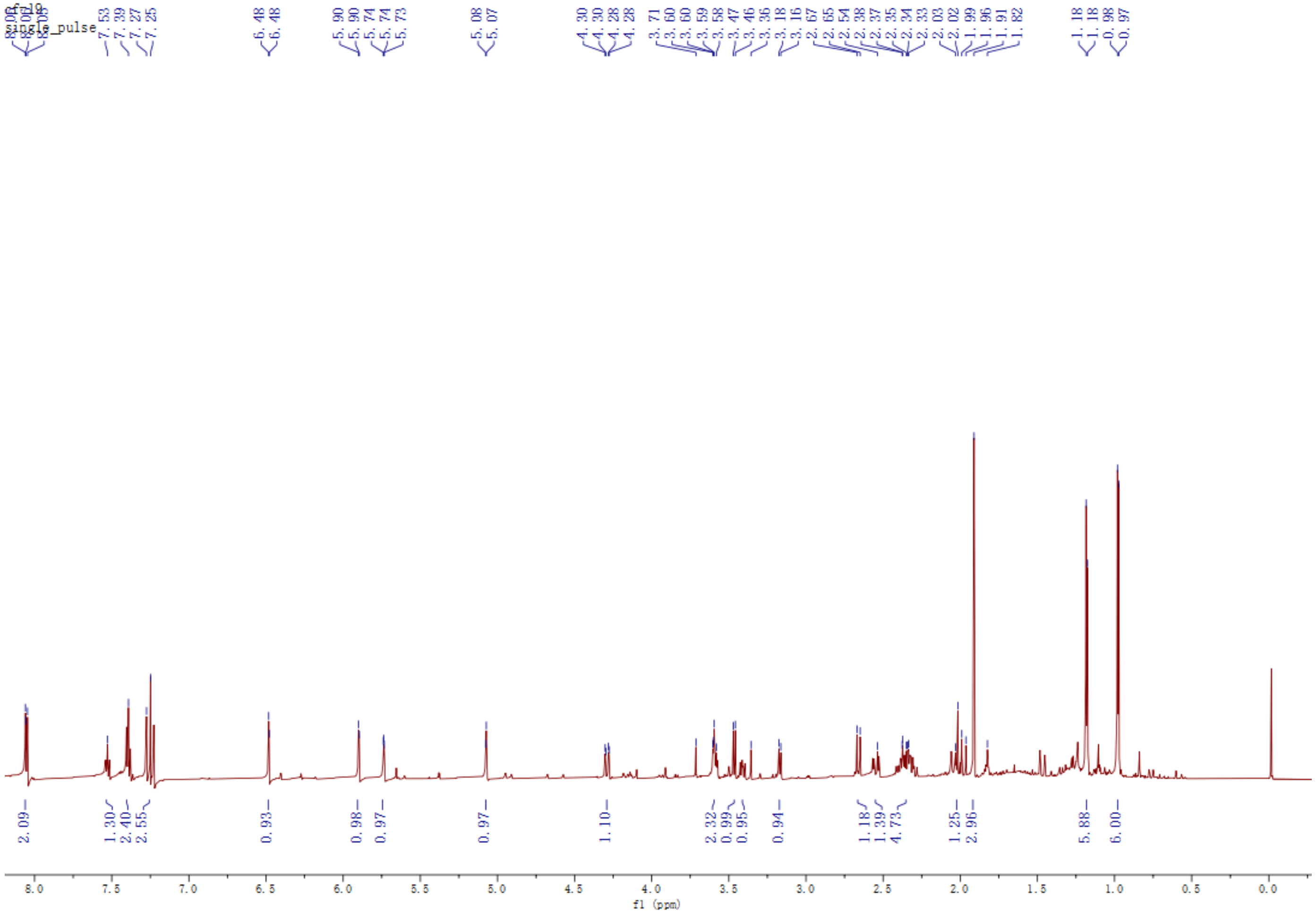

Figure S10. ^{1}H NMR spectrum (600 MHz) of compound **2** in $CDCl_3$.

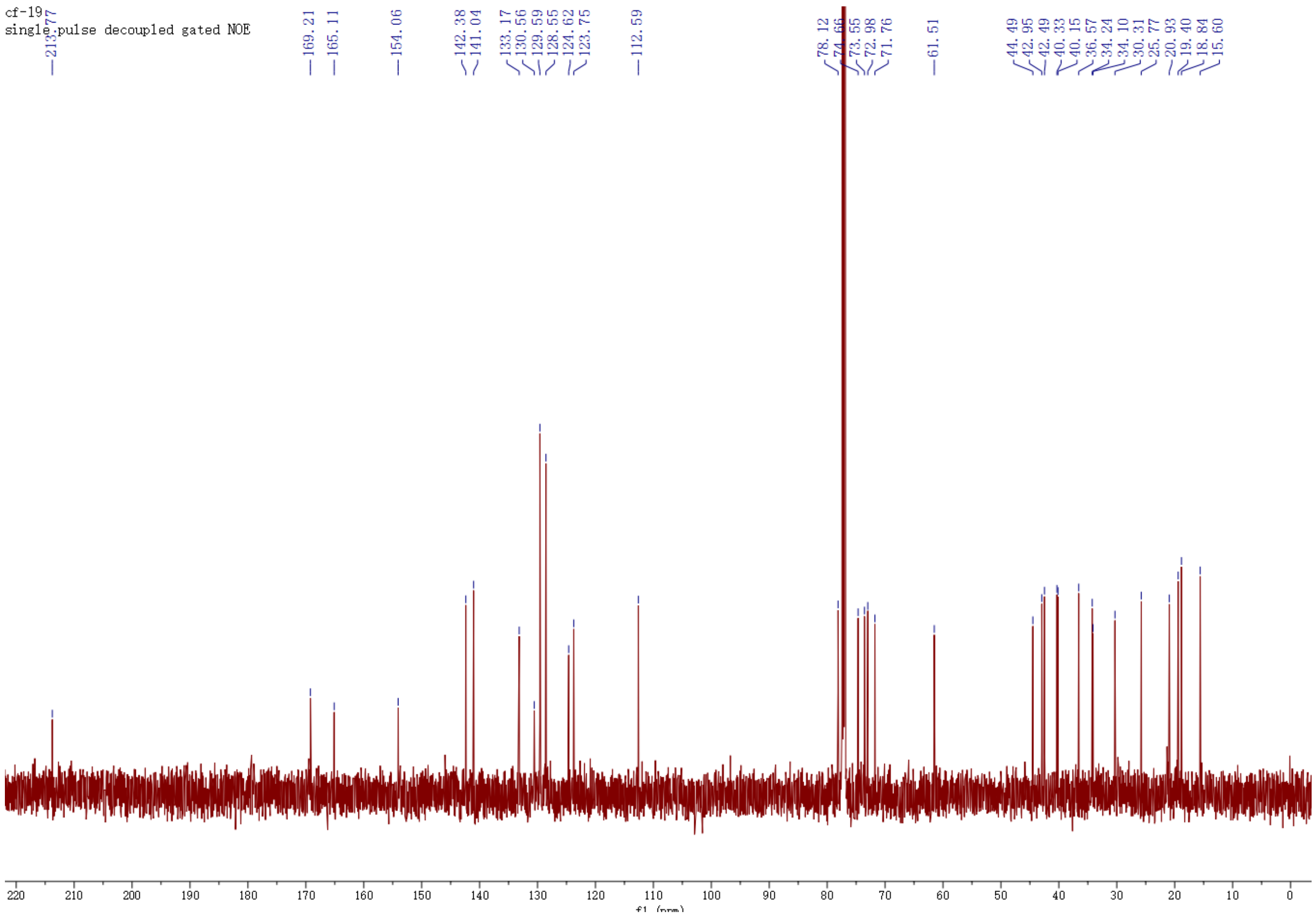

Figure S11. ^{13}C NMR spectrum (150 MHz) of compound **2** in $CDCl_3$.

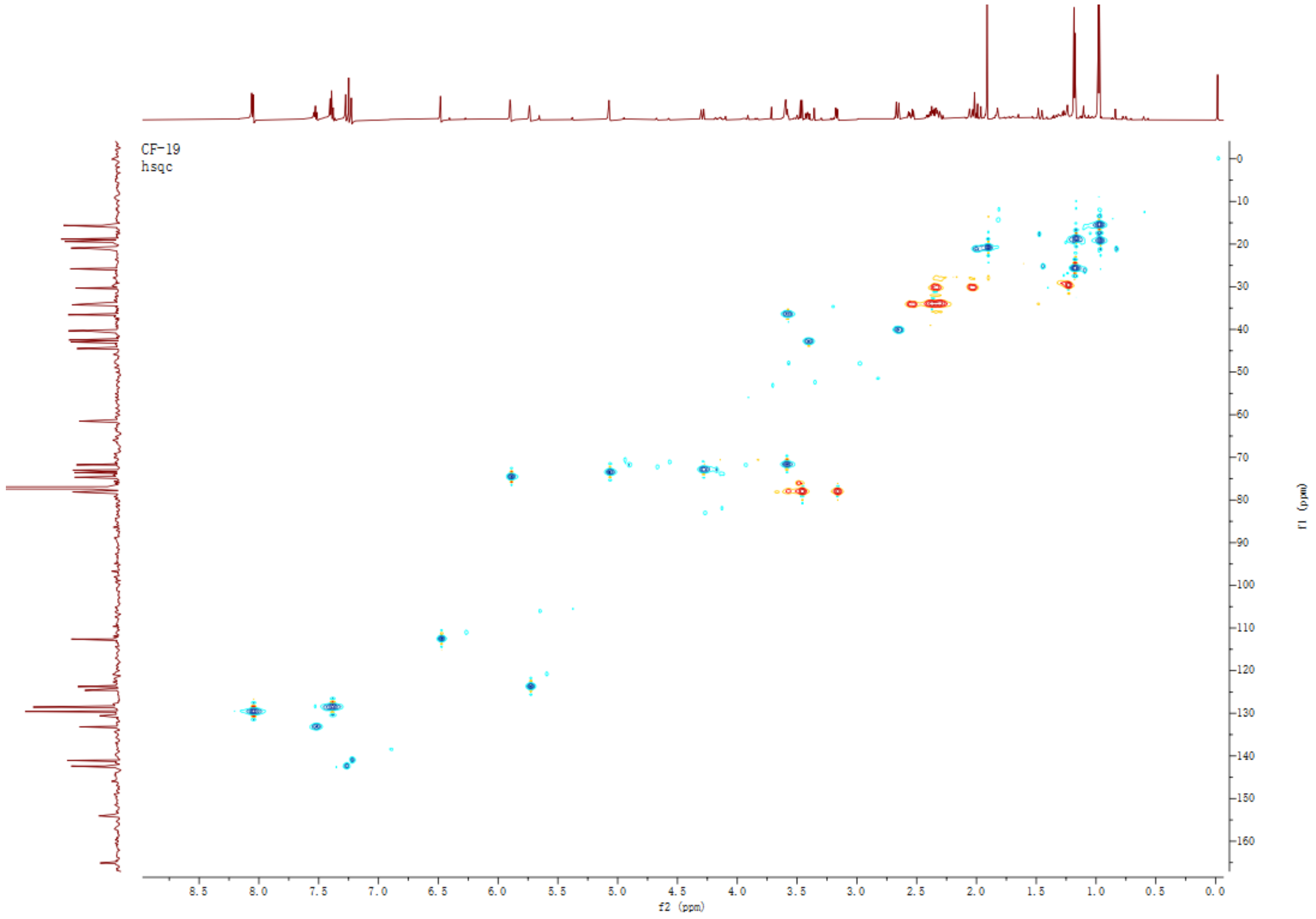

Figure S12. HSQC spectrum of compound **2** in $CDCl_3$.

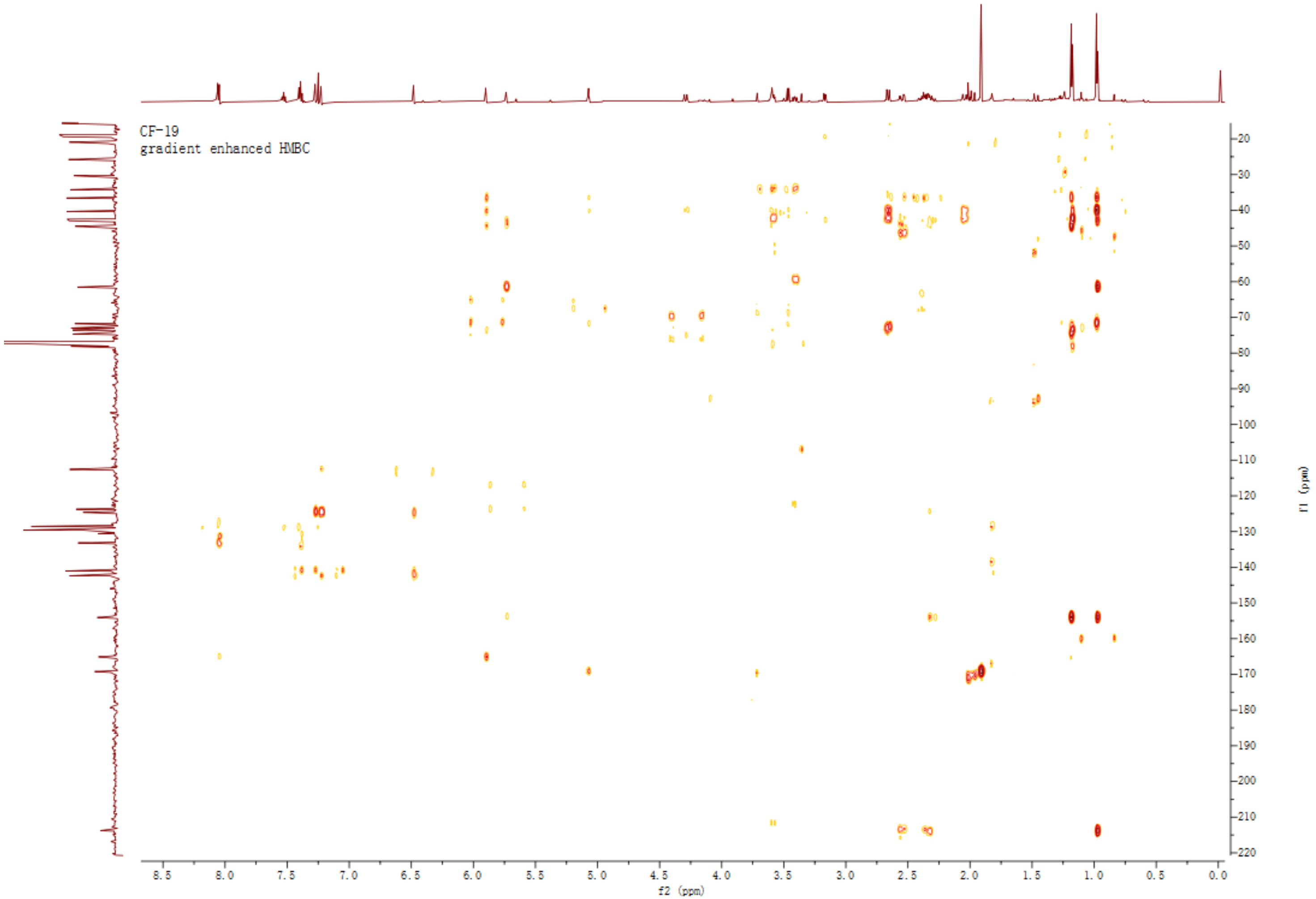

Figure S13. HMBC spectrum of compound **2** in $CDCl_3$.

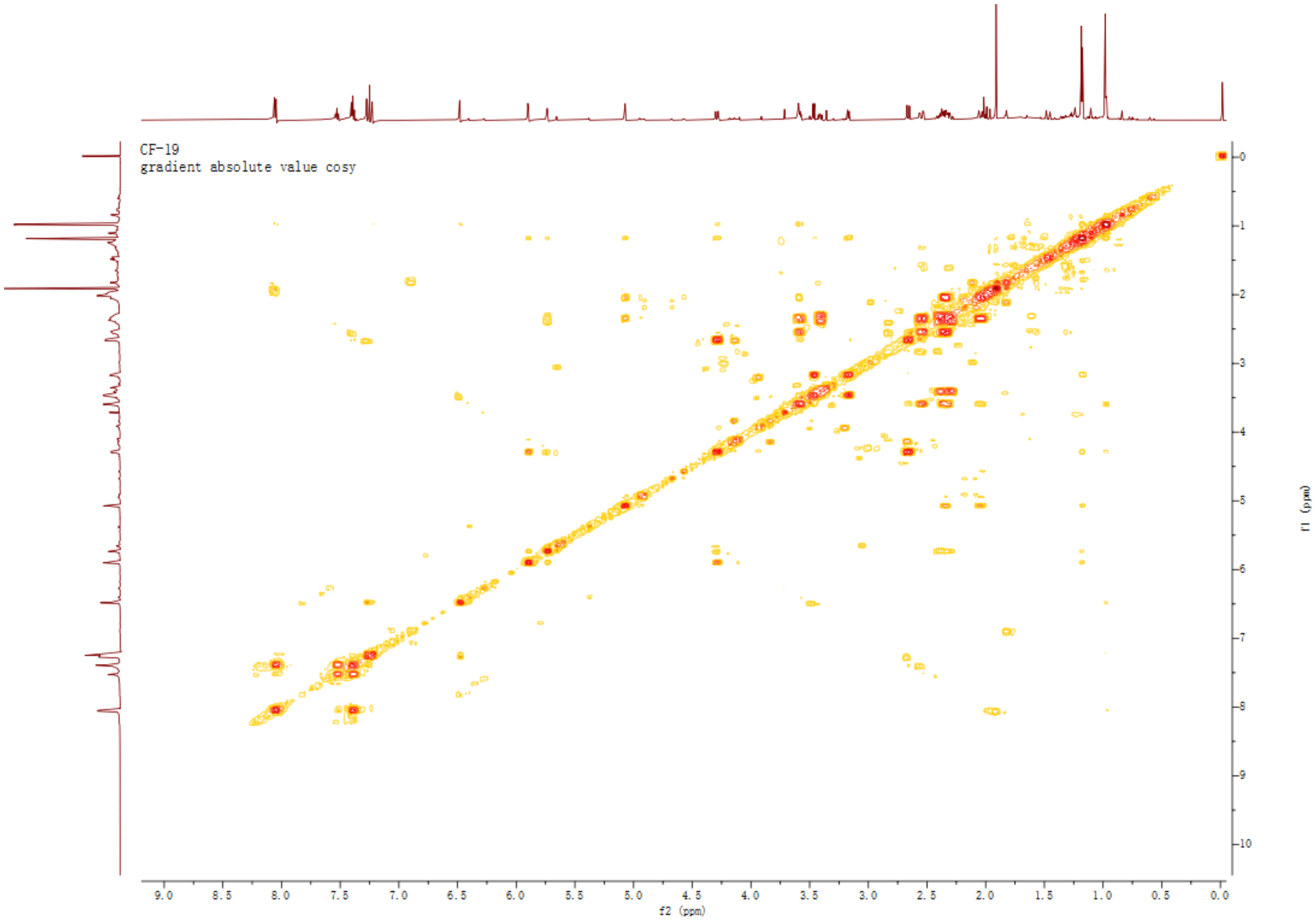

Figure S14. ^{1}H-^{1}H COSY spectrum of compound **2** in $CDCl_3$.

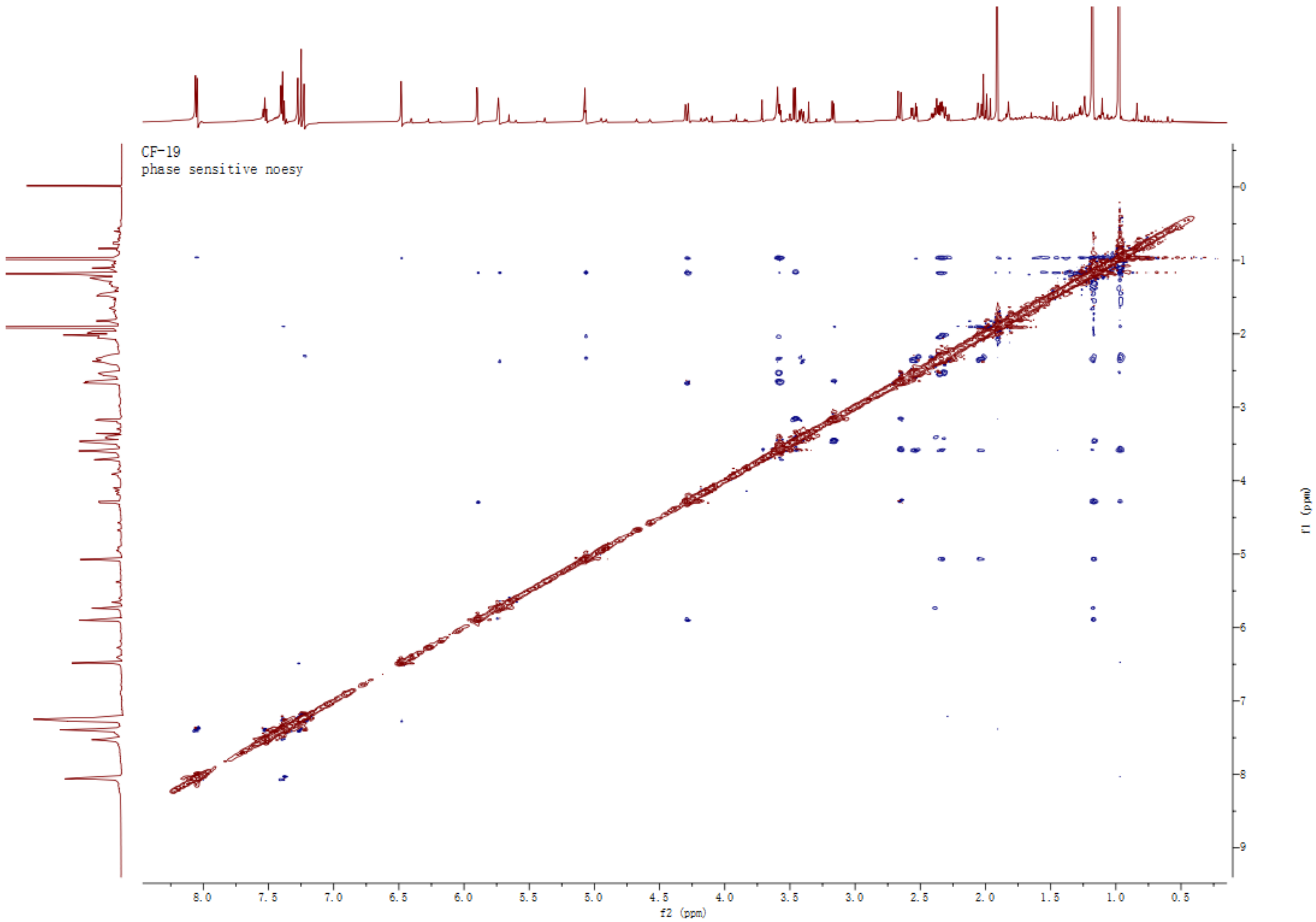

Figure S15. NOESY spectrum of Compound **2** in $CDCl_3$

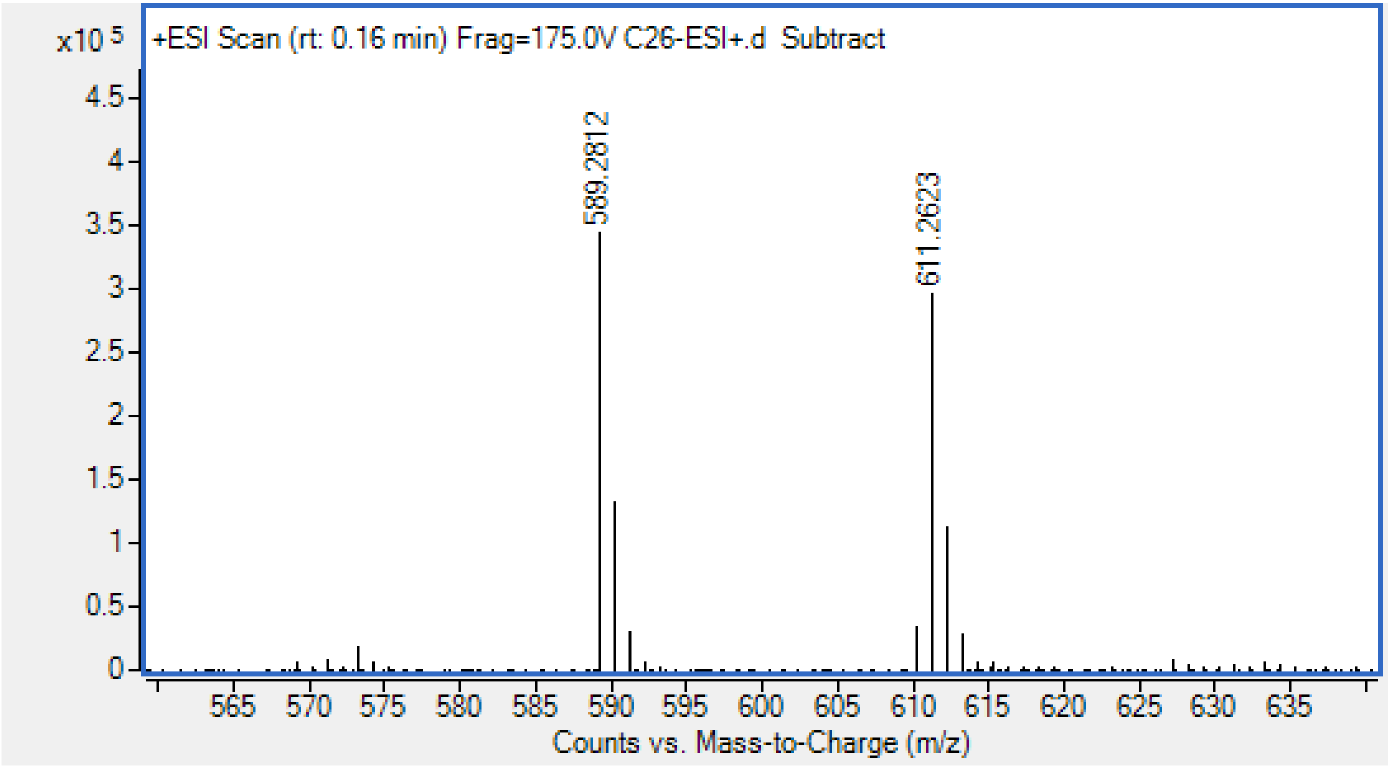

Figure S16. HRESIMS spectrum of compound **2**

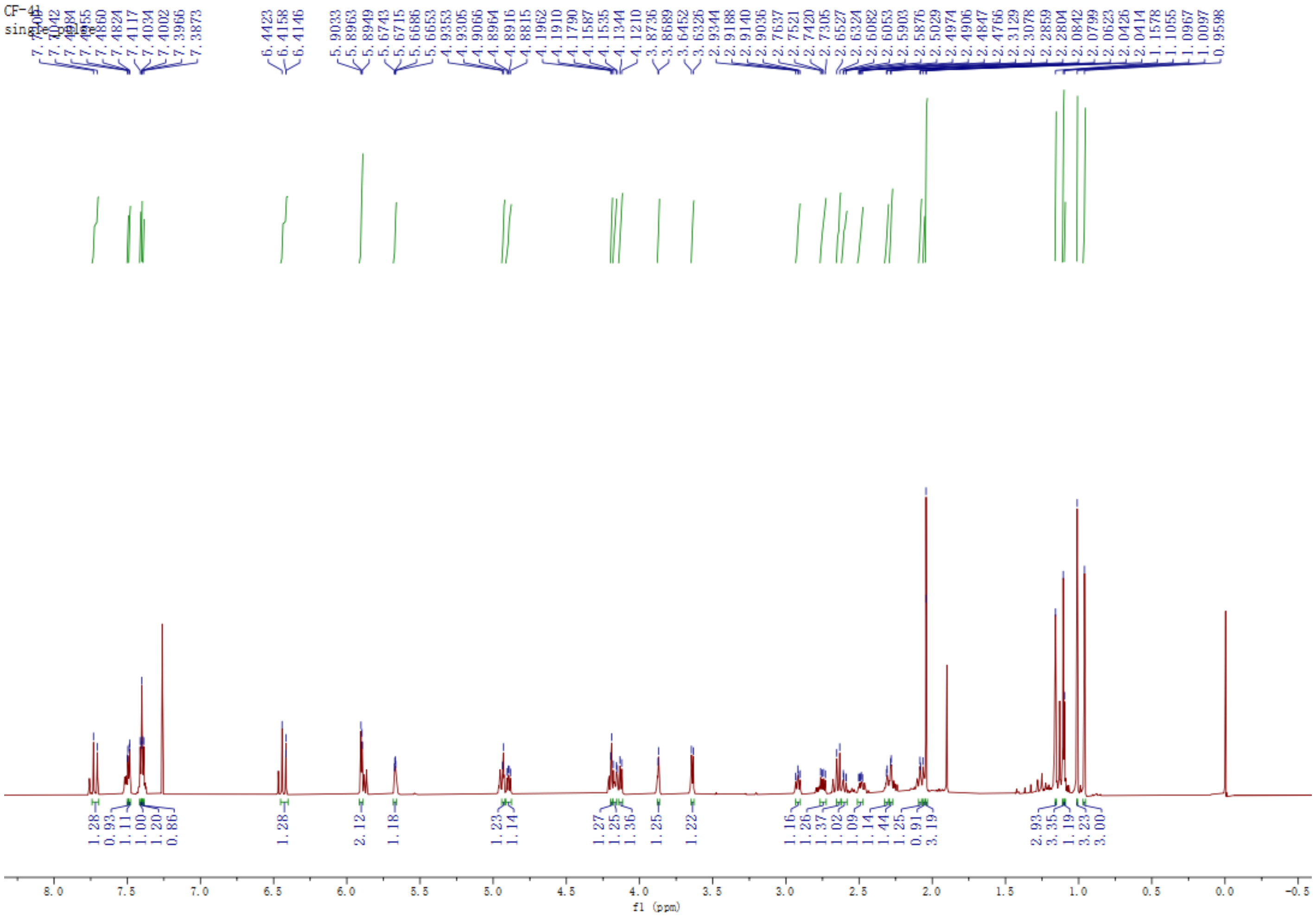

Figure S17. ^{1}H NMR spectrum (600 MHz) of compound **3** in $CDCl_3$.

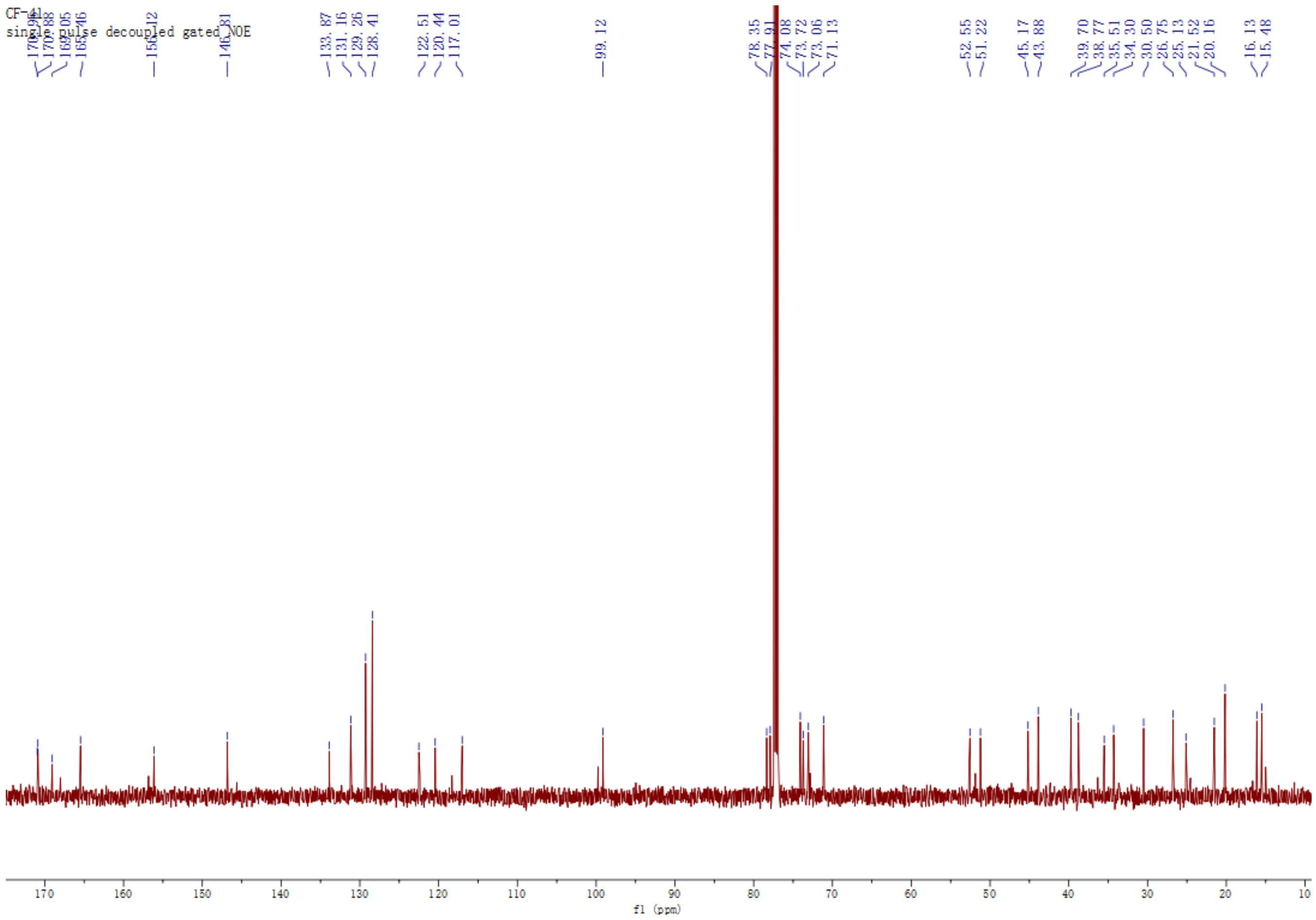

Figure S18. ^{13}C NMR spectrum (150 MHz) of compound **3** in $CDCl_3$.

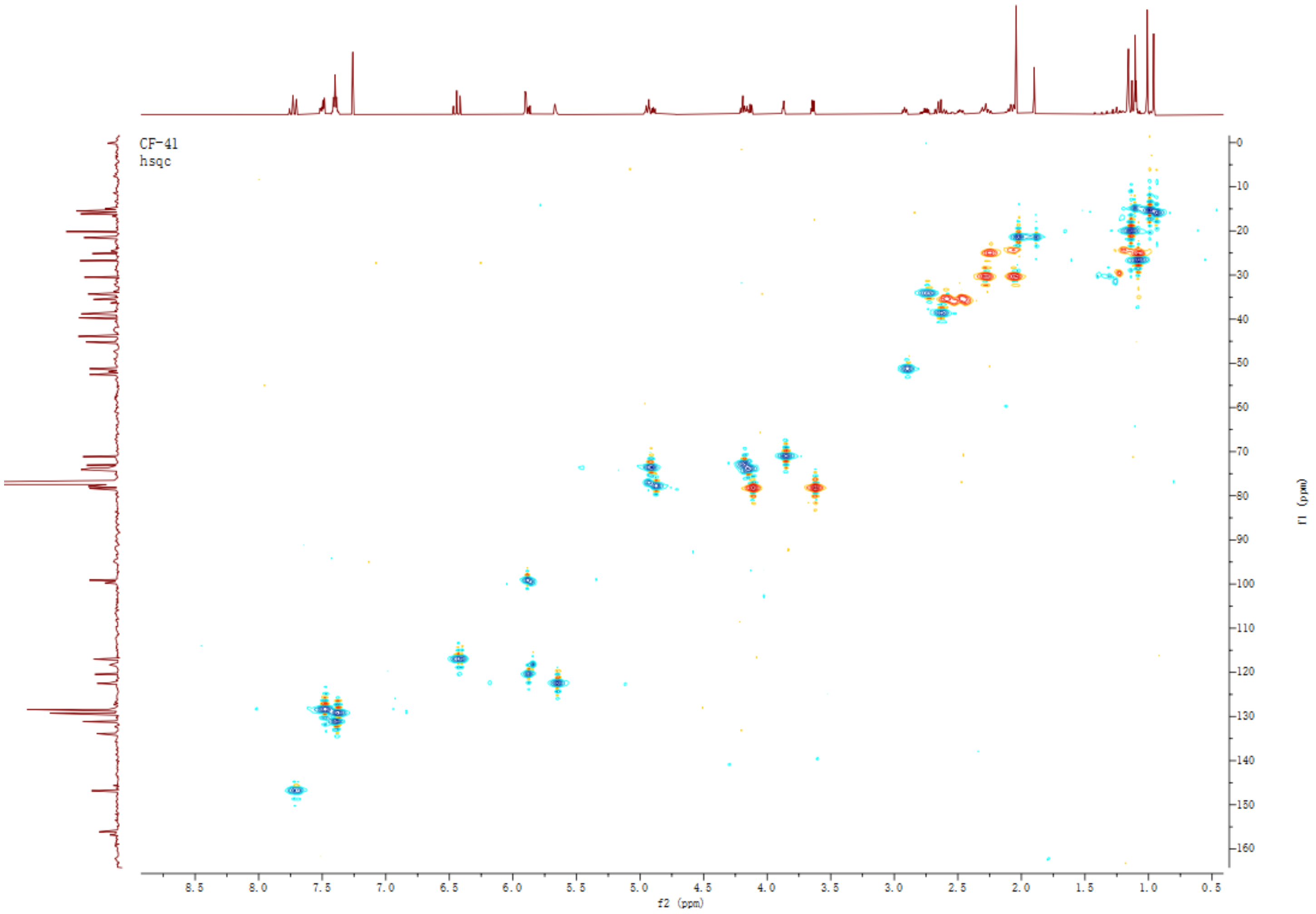

Figure S19. HSQC spectrum of compound **3** in $CDCl_3$.

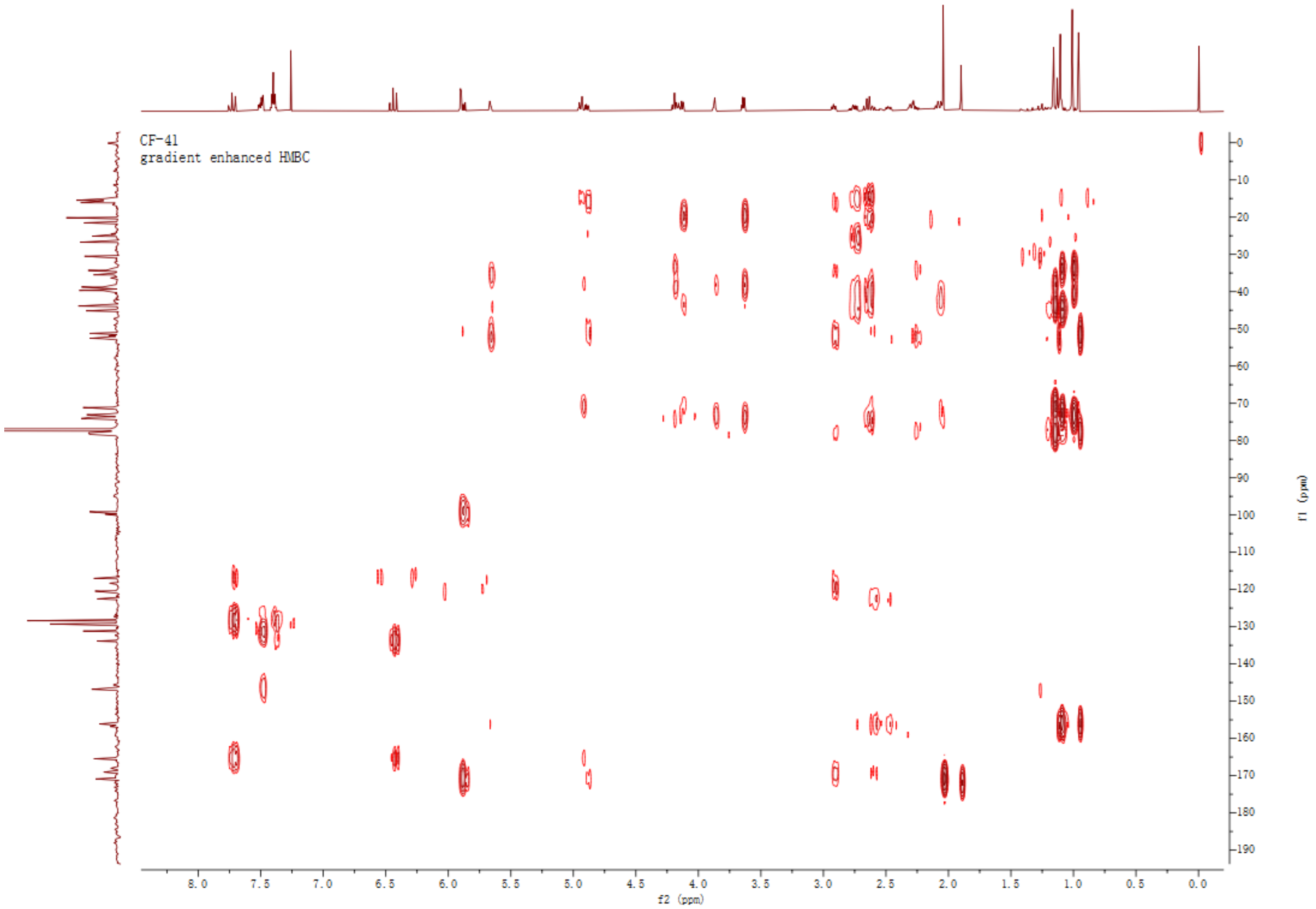

Figure S20. HMBC spectrum of compound **3** in $CDCl_3$.

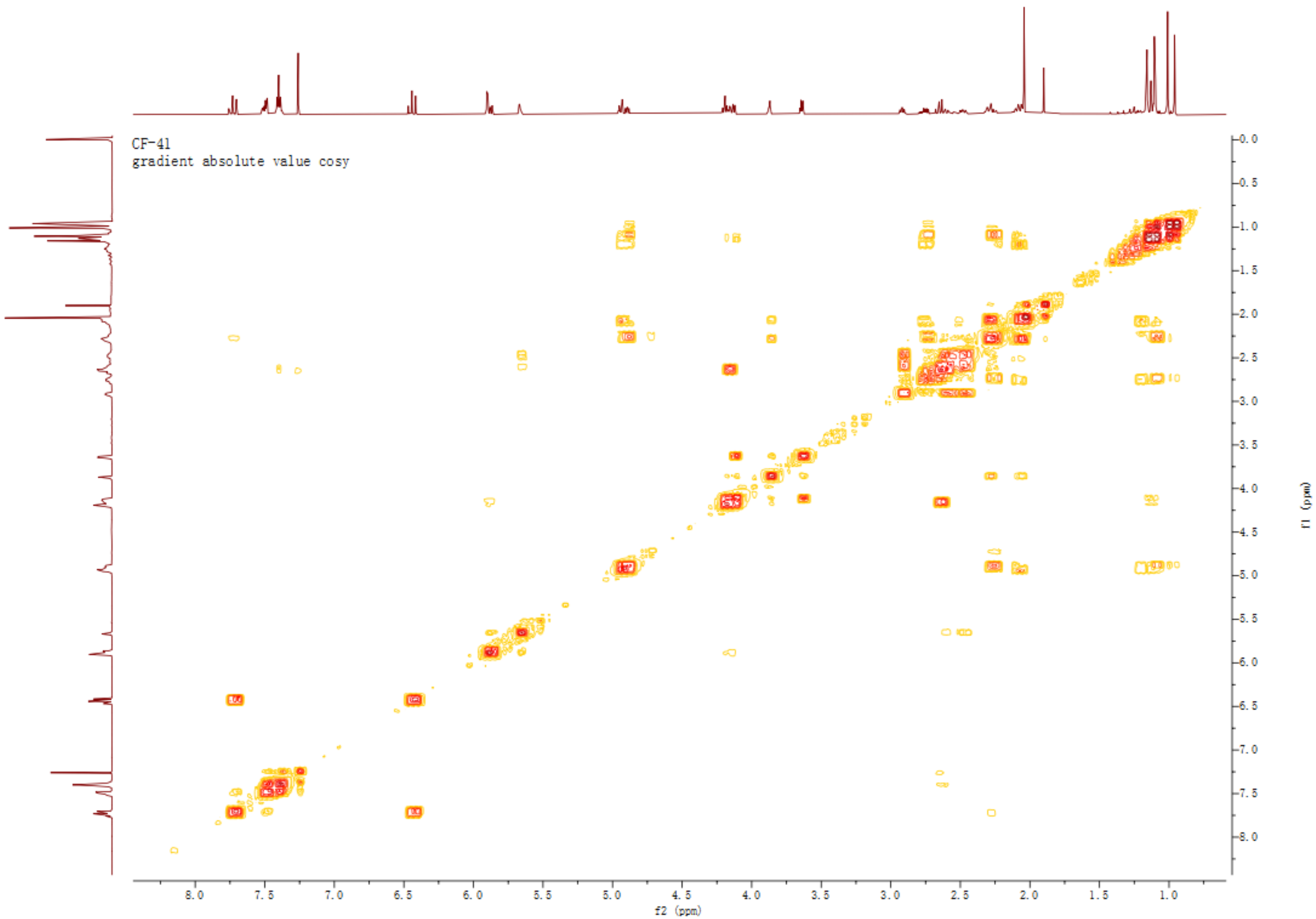

Figure S21. ^{1}H-^{1}H COSY spectrum of compound **3** in $CDCl_3$.

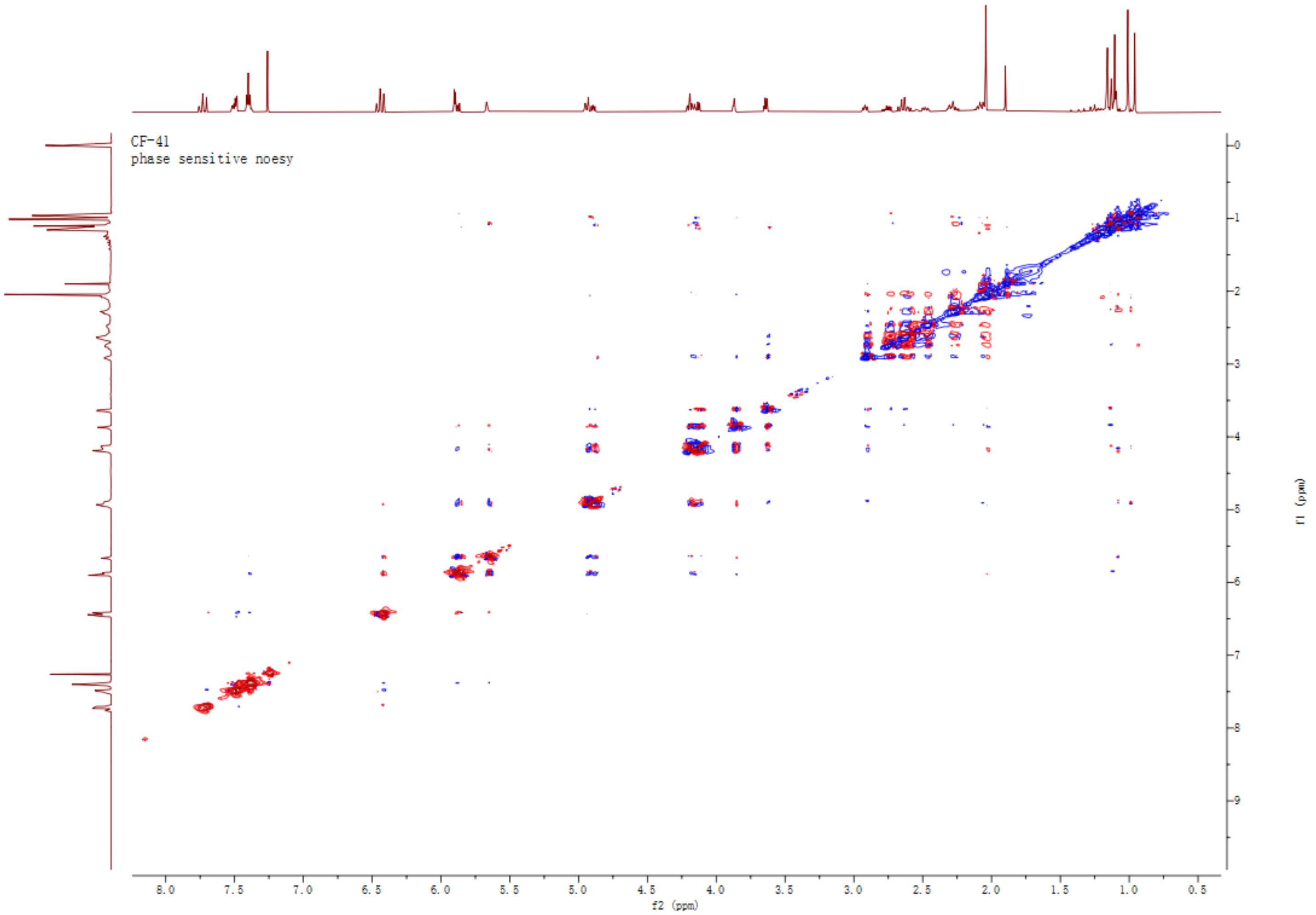

Figure S22. NOESY spectrum of Compound **3** in $CDCl_3$

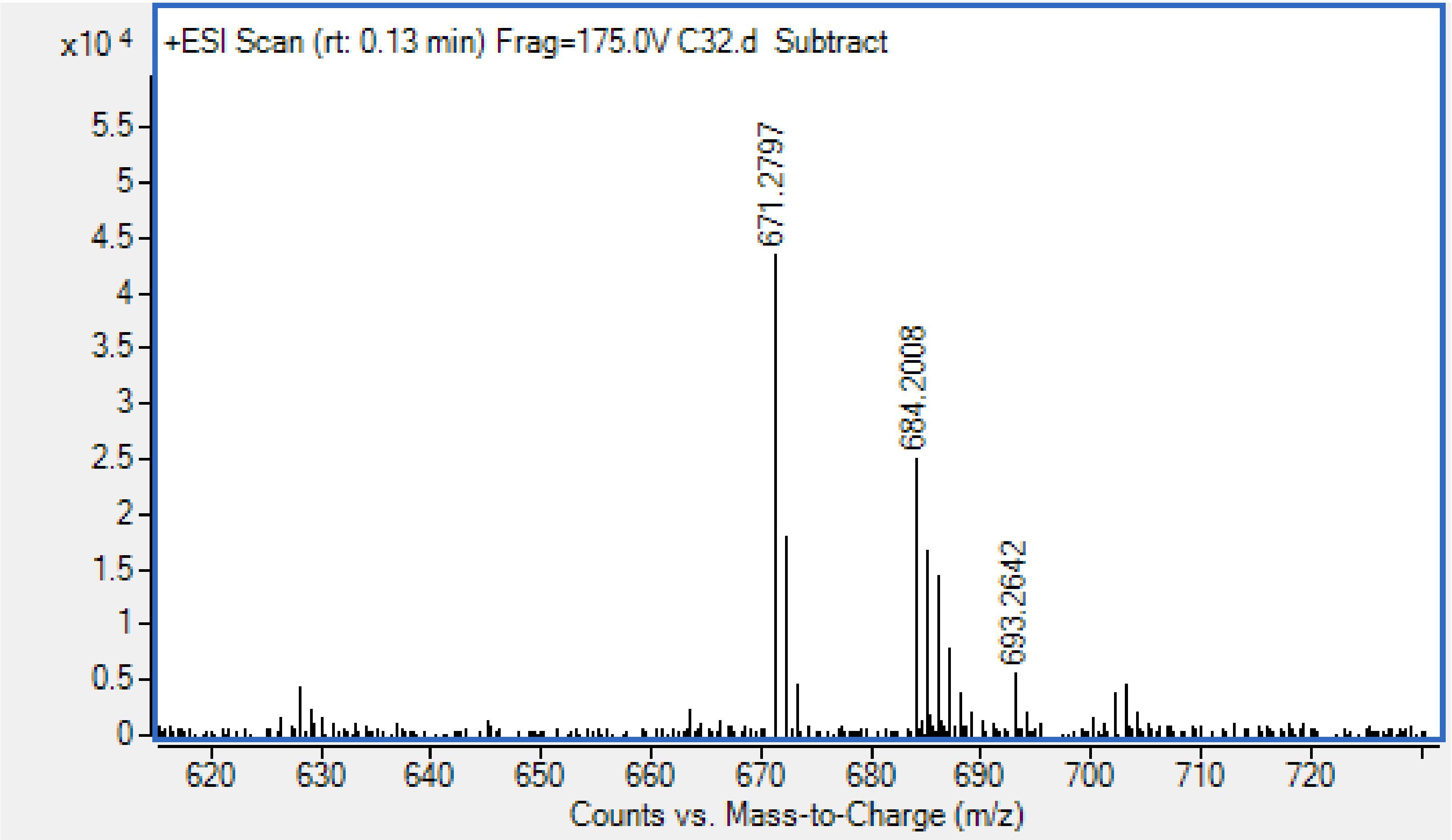

Figure S23. HRESIMS spectrum of compound **3**.

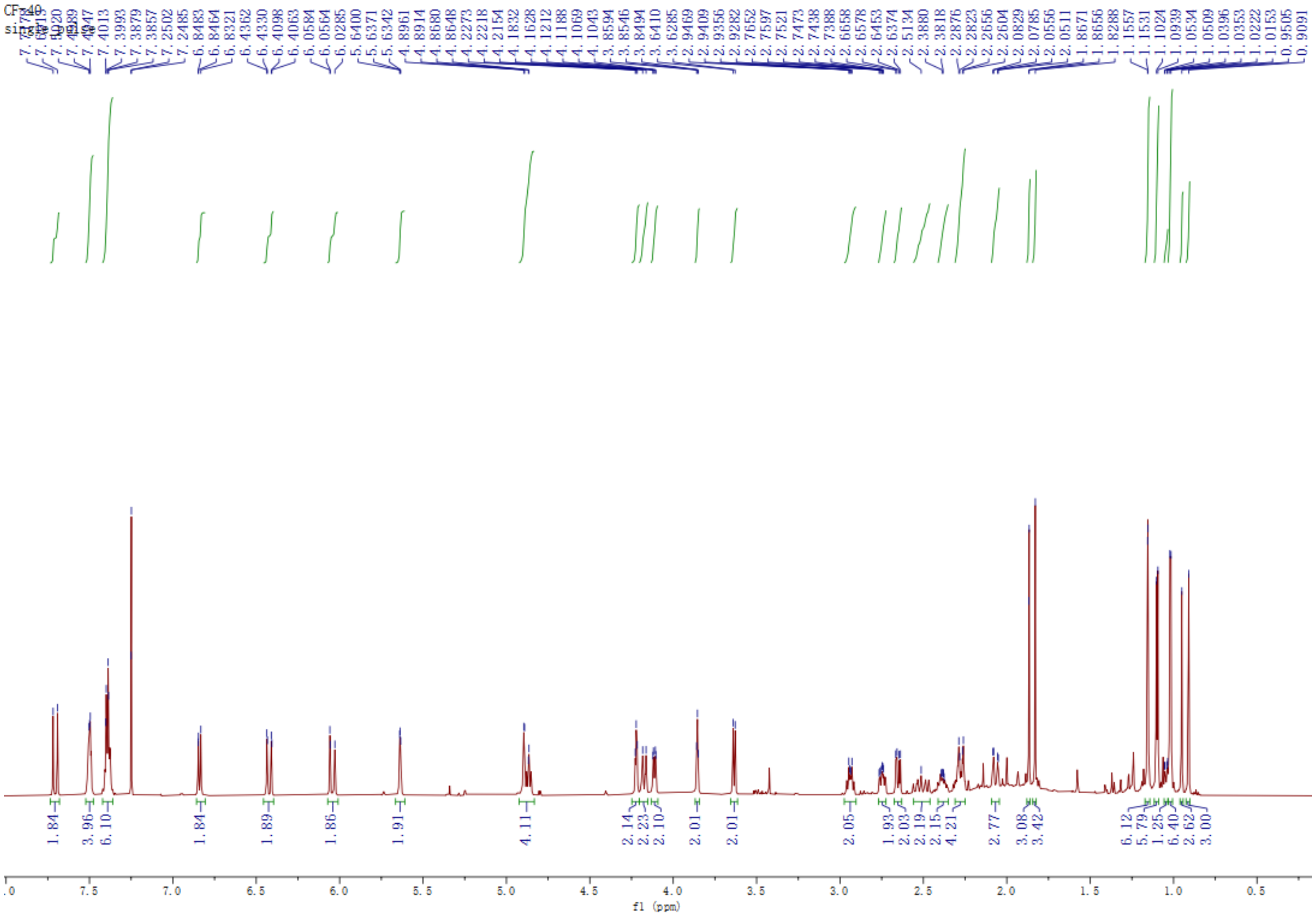

Figure S24. ^{1}H NMR spectrum (600 MHz) of compound **4** in $CDCl_3$.

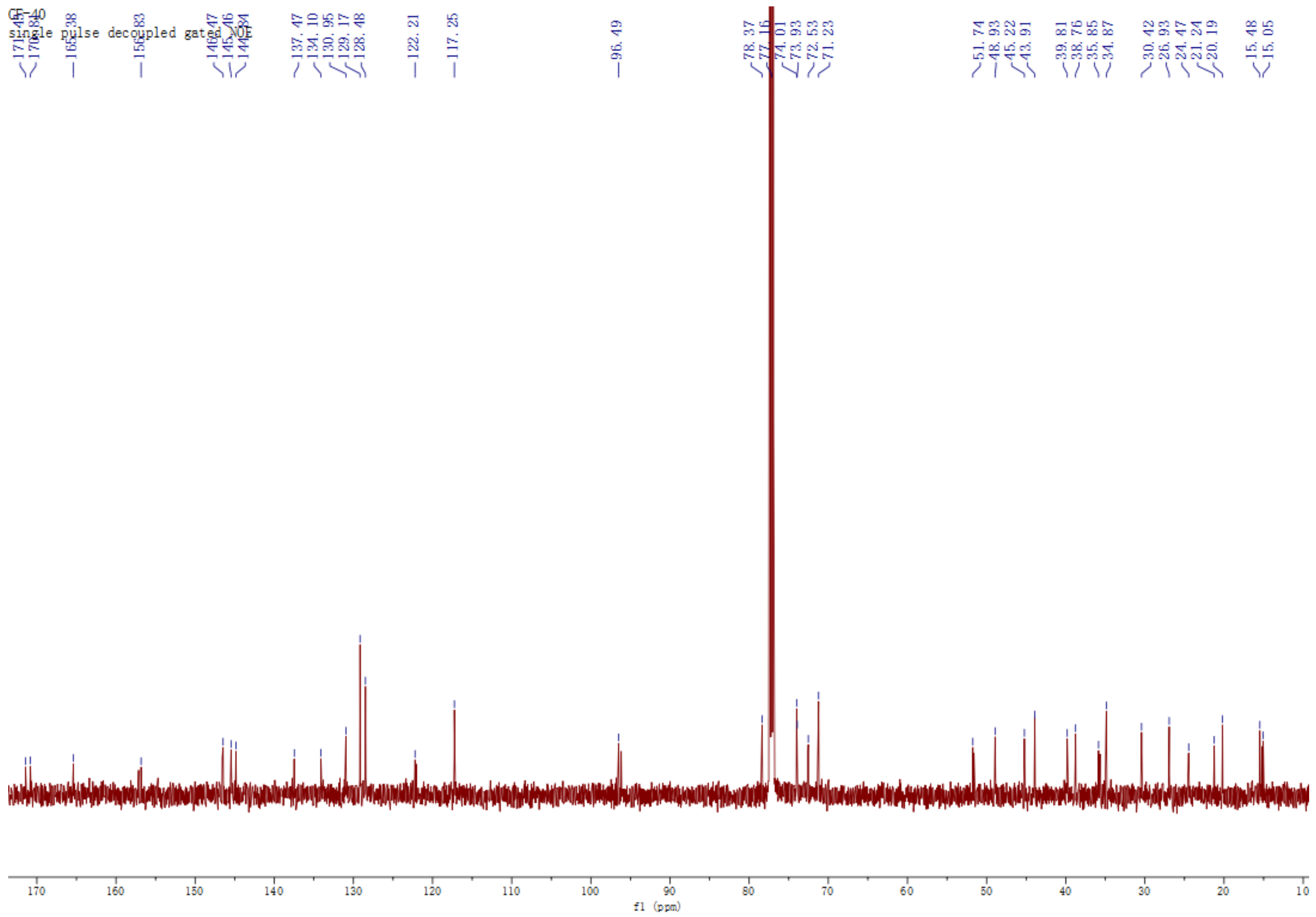

Figure S25. ^{13}C NMR spectrum (150 MHz) of compound **4** in $CDCl_3$.

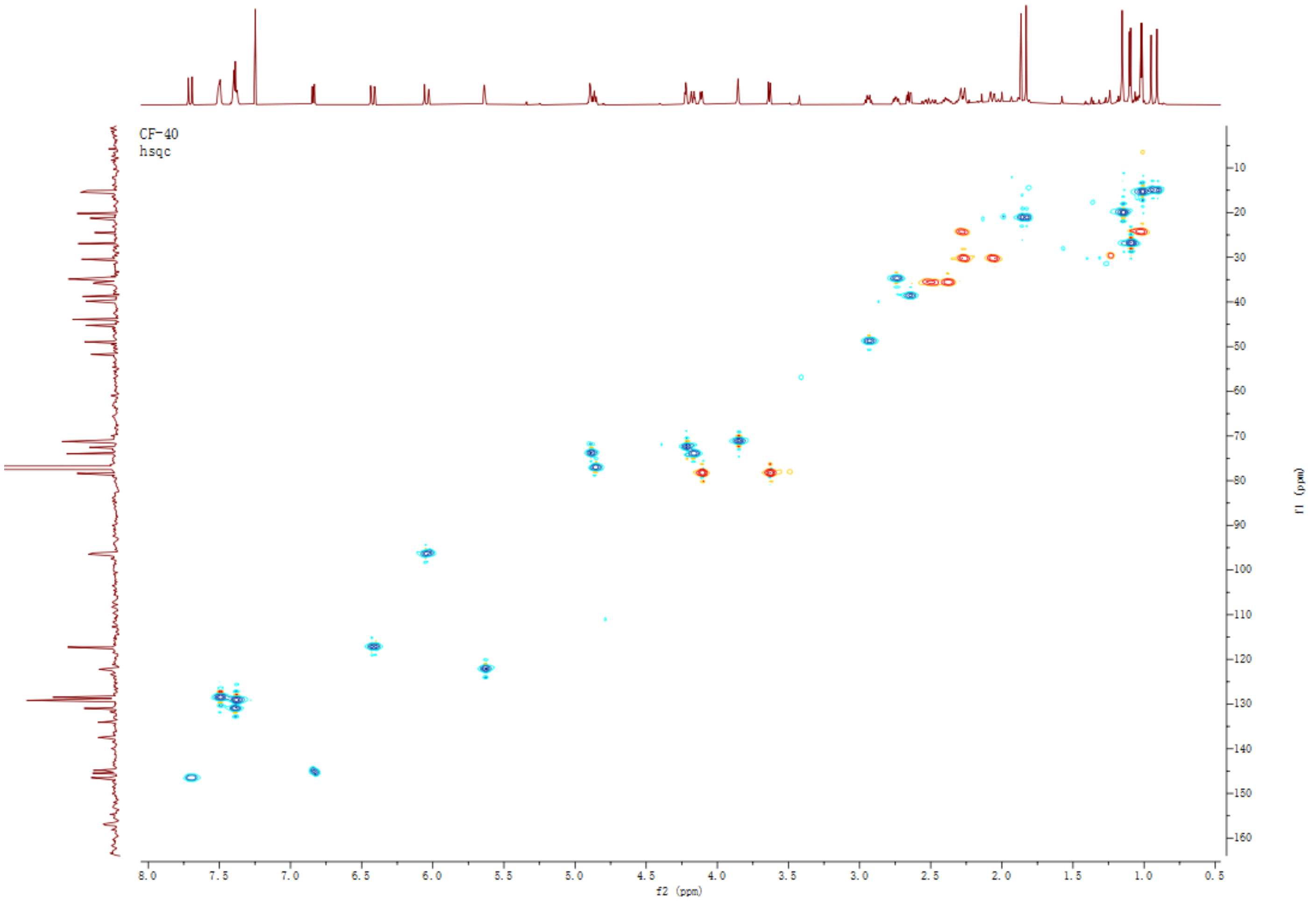

Figure S26. HSQC spectrum of compound **4** in $CDCl_3$.

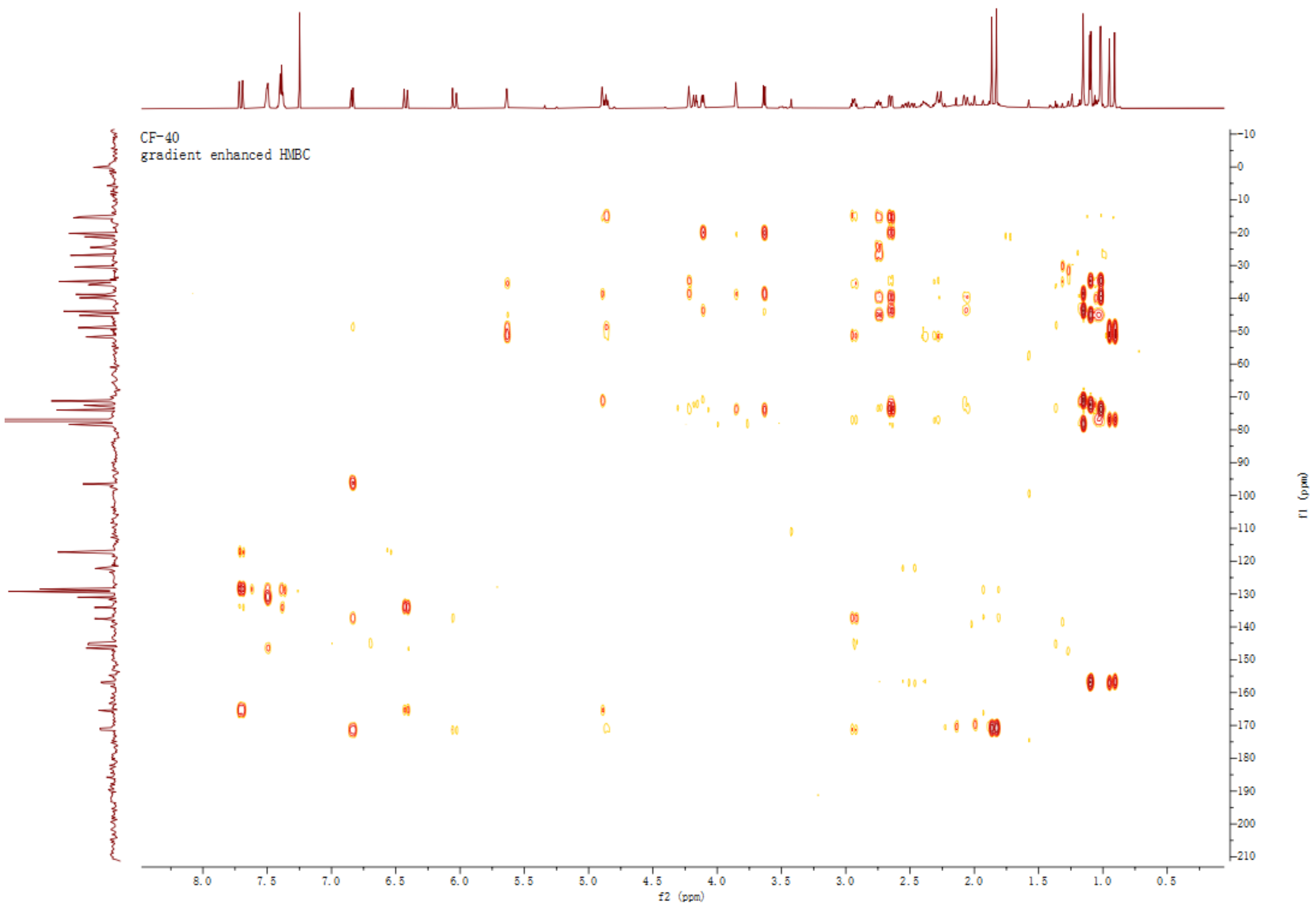

Figure S27. HMBC spectrum of compound **4** in $CDCl_3$.

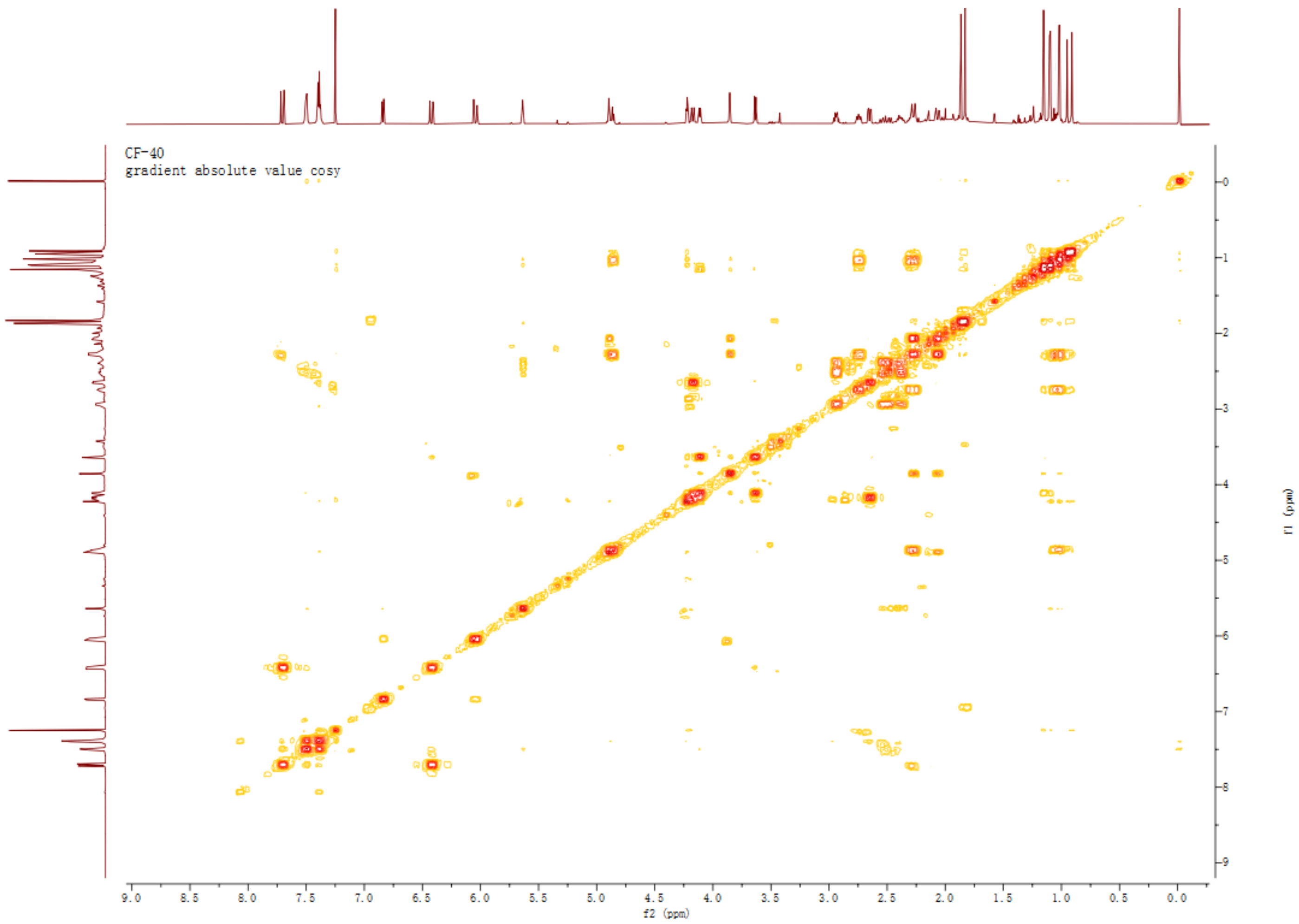

Figure S28. ^{1}H-^{1}H COSY spectrum of compound **4** in $CDCl_3$.

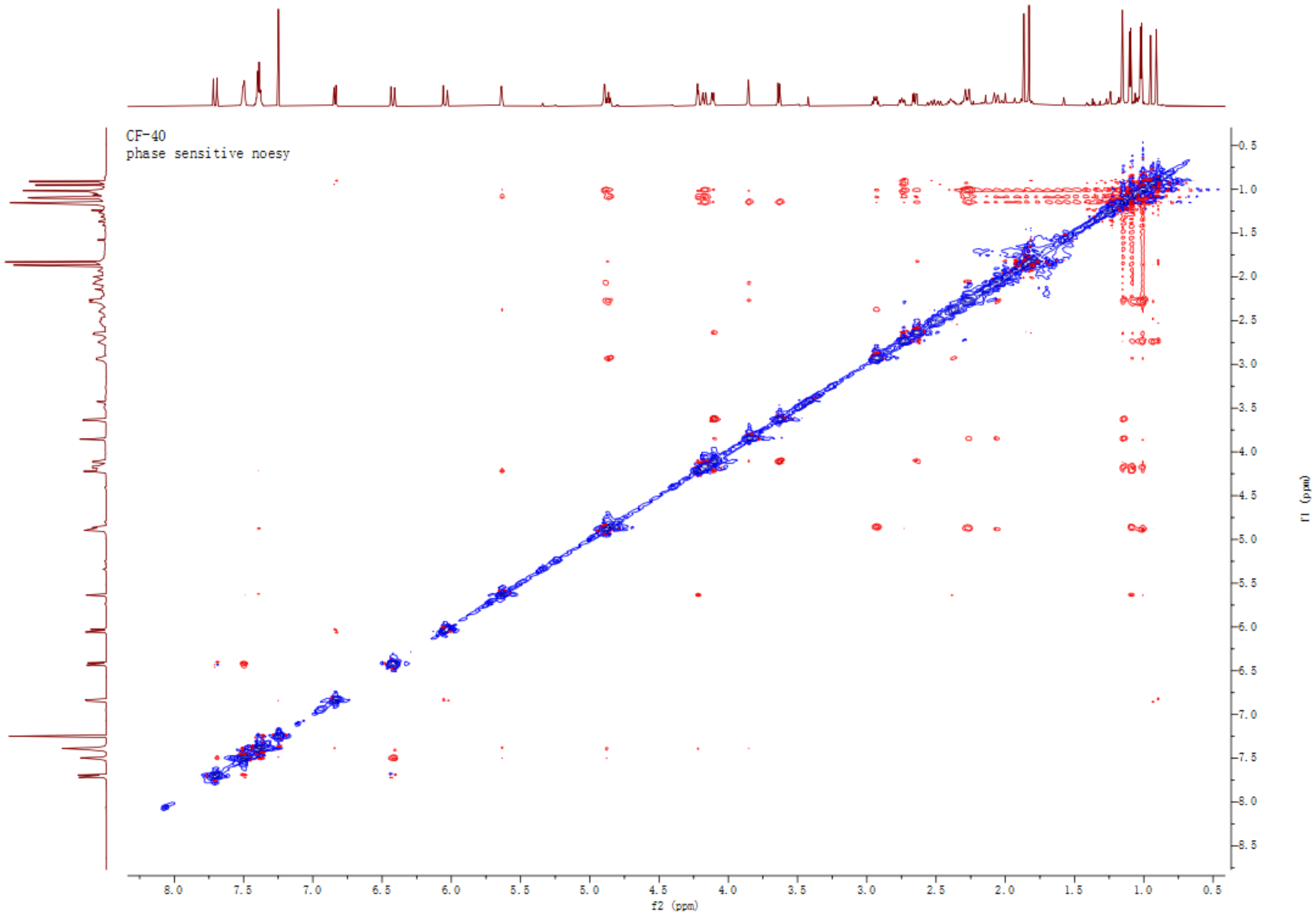

Figure S29. NOESY spectrum of Compound **4** in $CDCl_3$

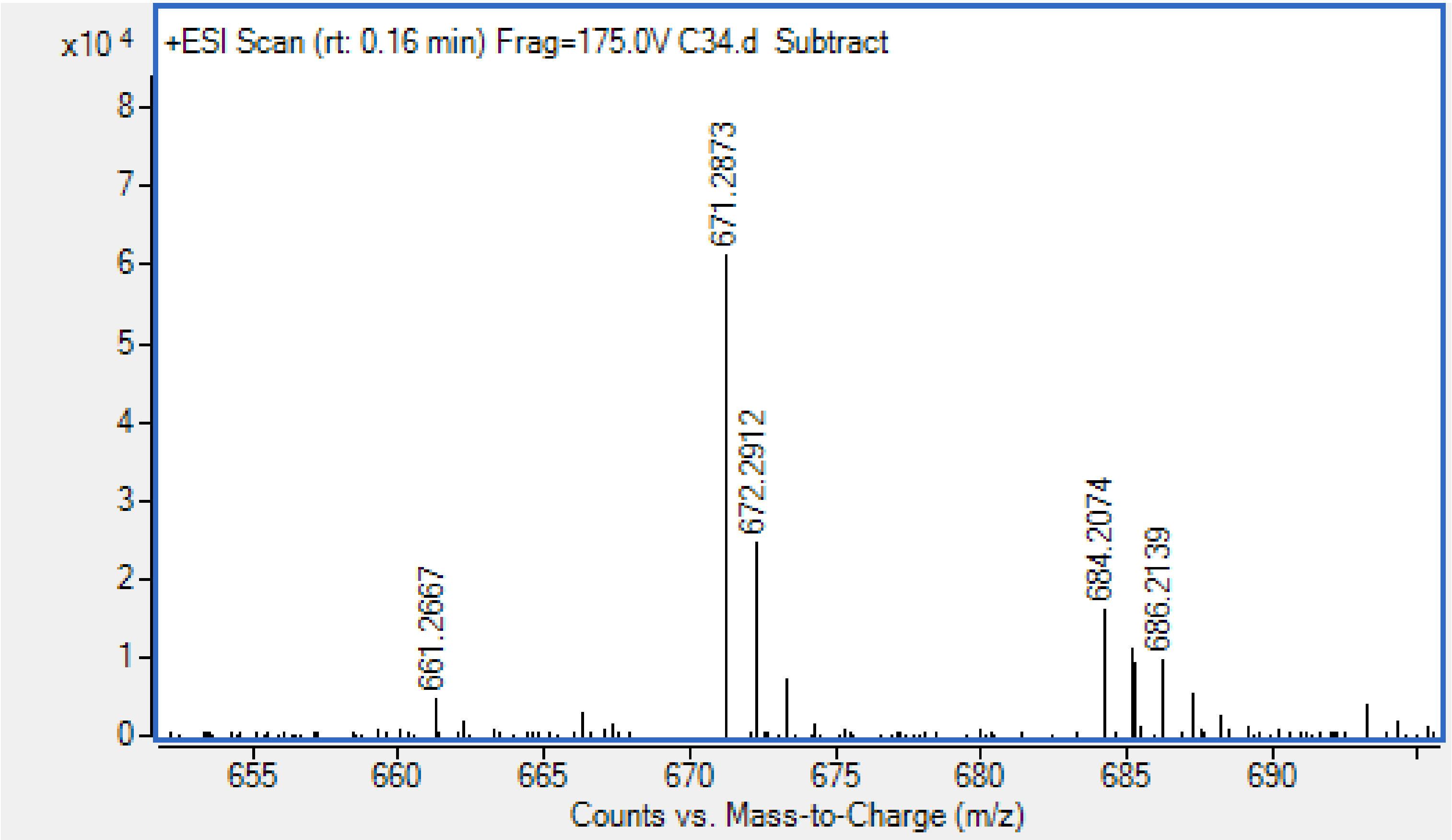

Figure S30. HRESIMS spectrum of compound **4**.

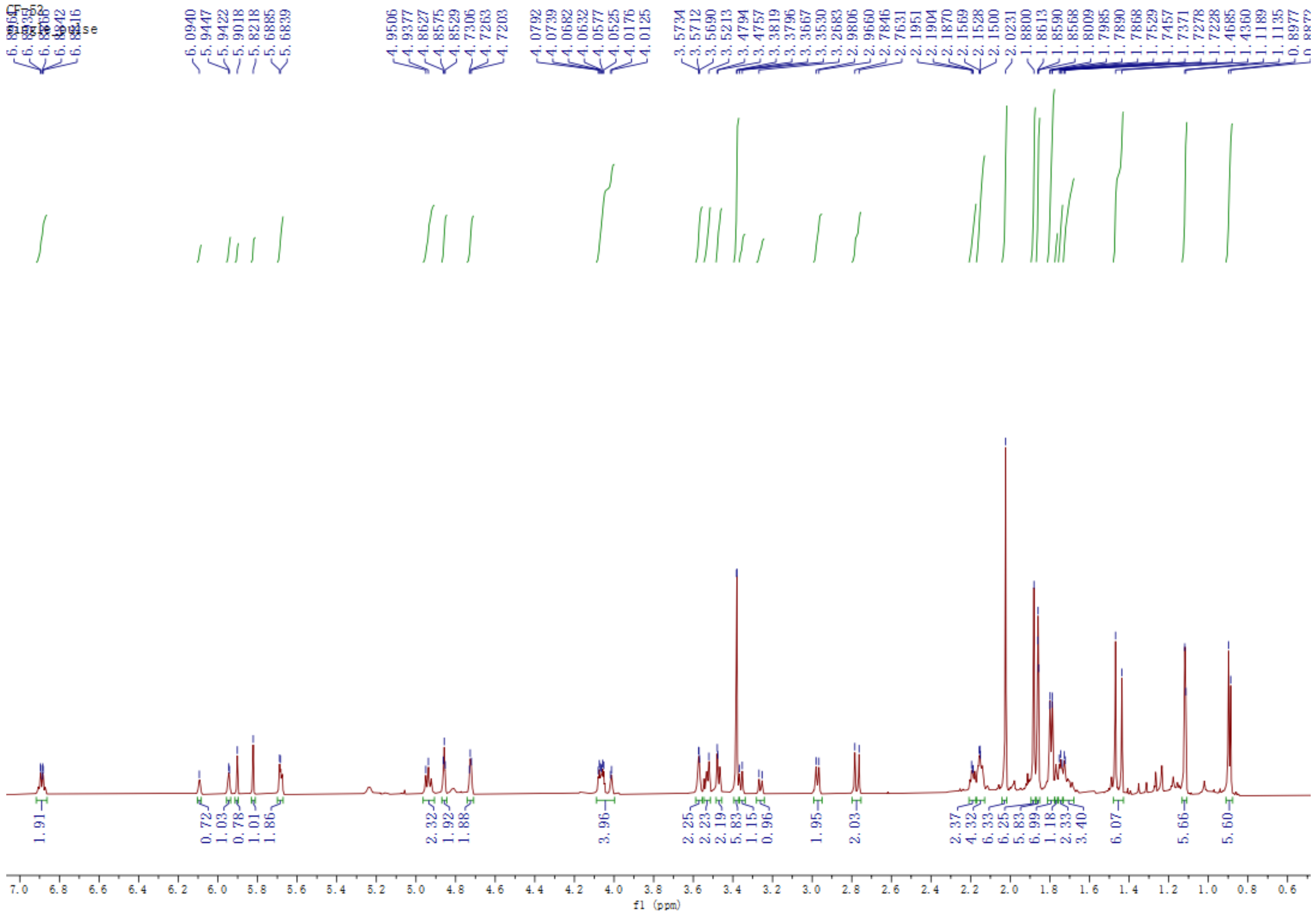

Figure S31. ^{1}H NMR spectrum (600 MHz) of compound **5** in $CDCl_3$.

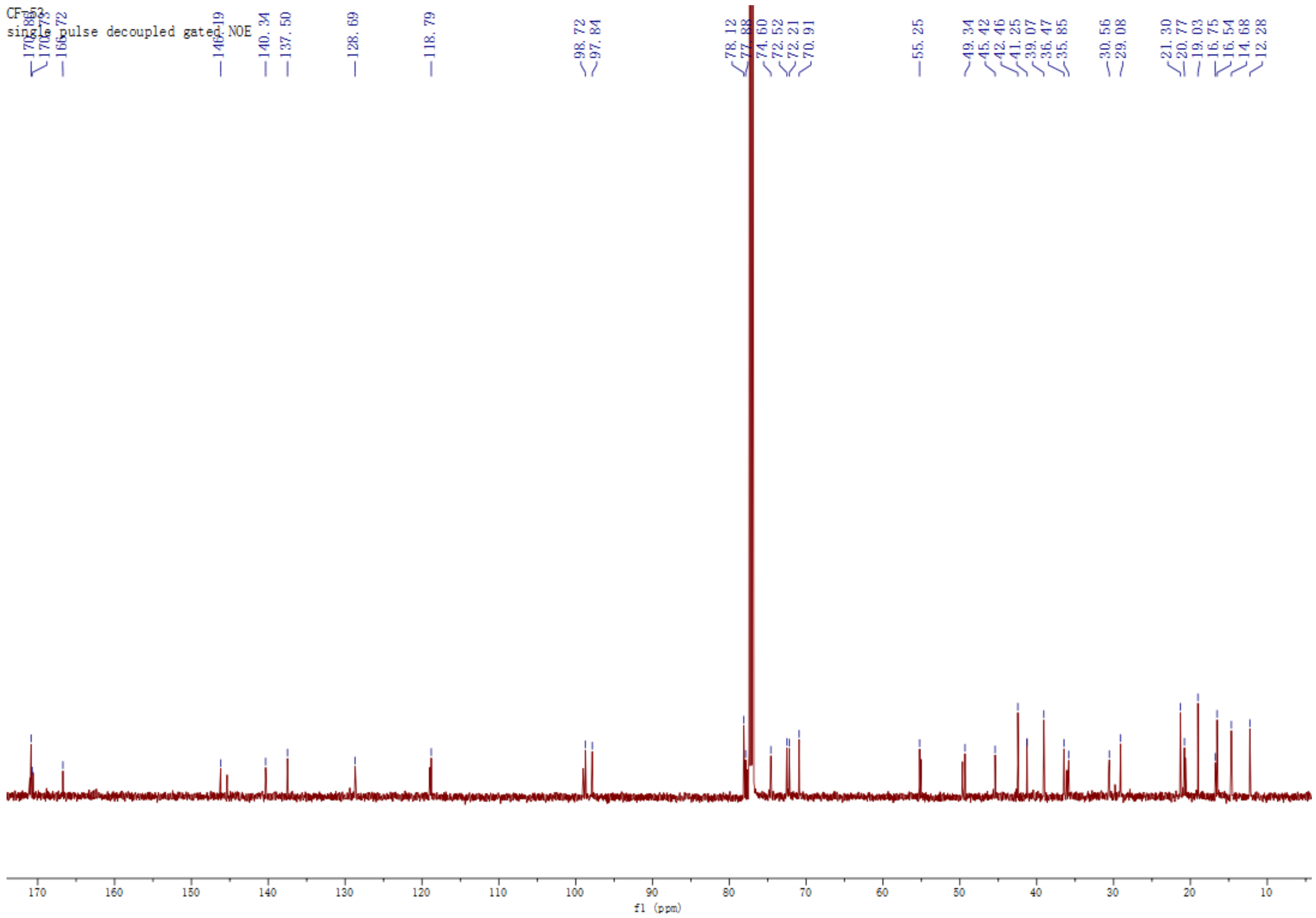

Figure S32. ^{13}C NMR spectrum (150 MHz) of compound **5** in $CDCl_3$.

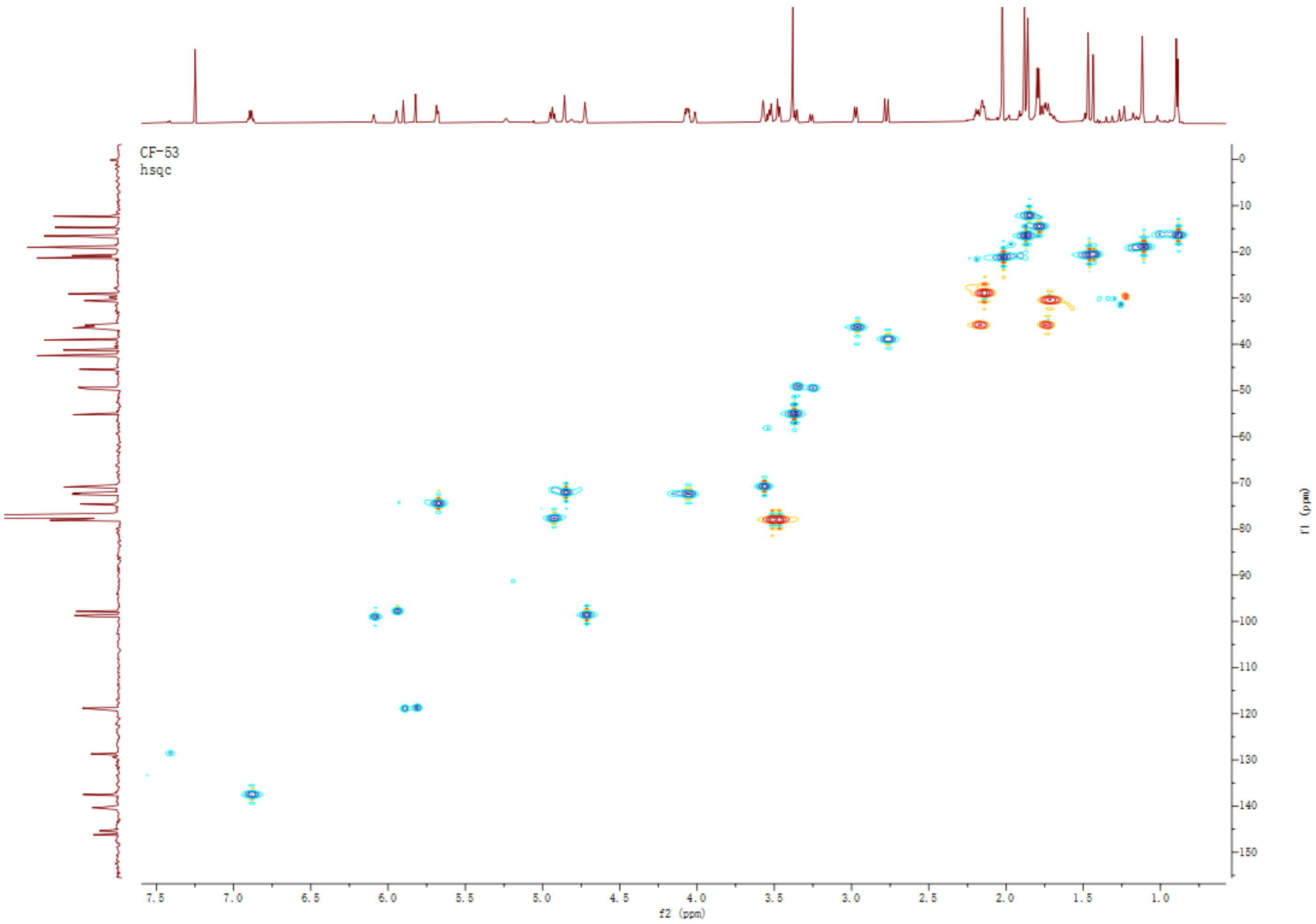

Figure S33. HSQC spectrum of compound **5** in $CDCl_3$.

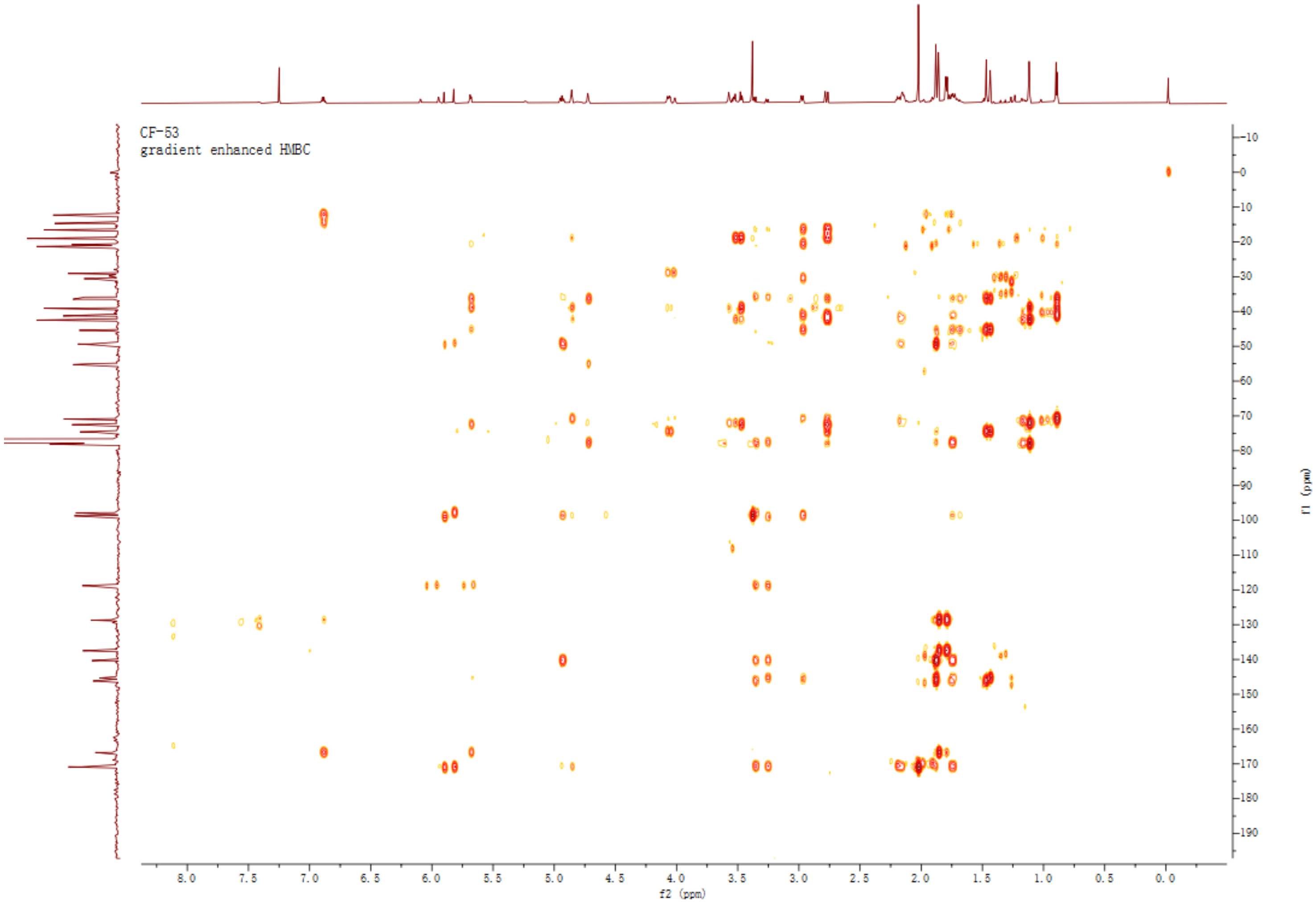

Figure S34. HMBC spectrum of compound **5** in $CDCl_3$.

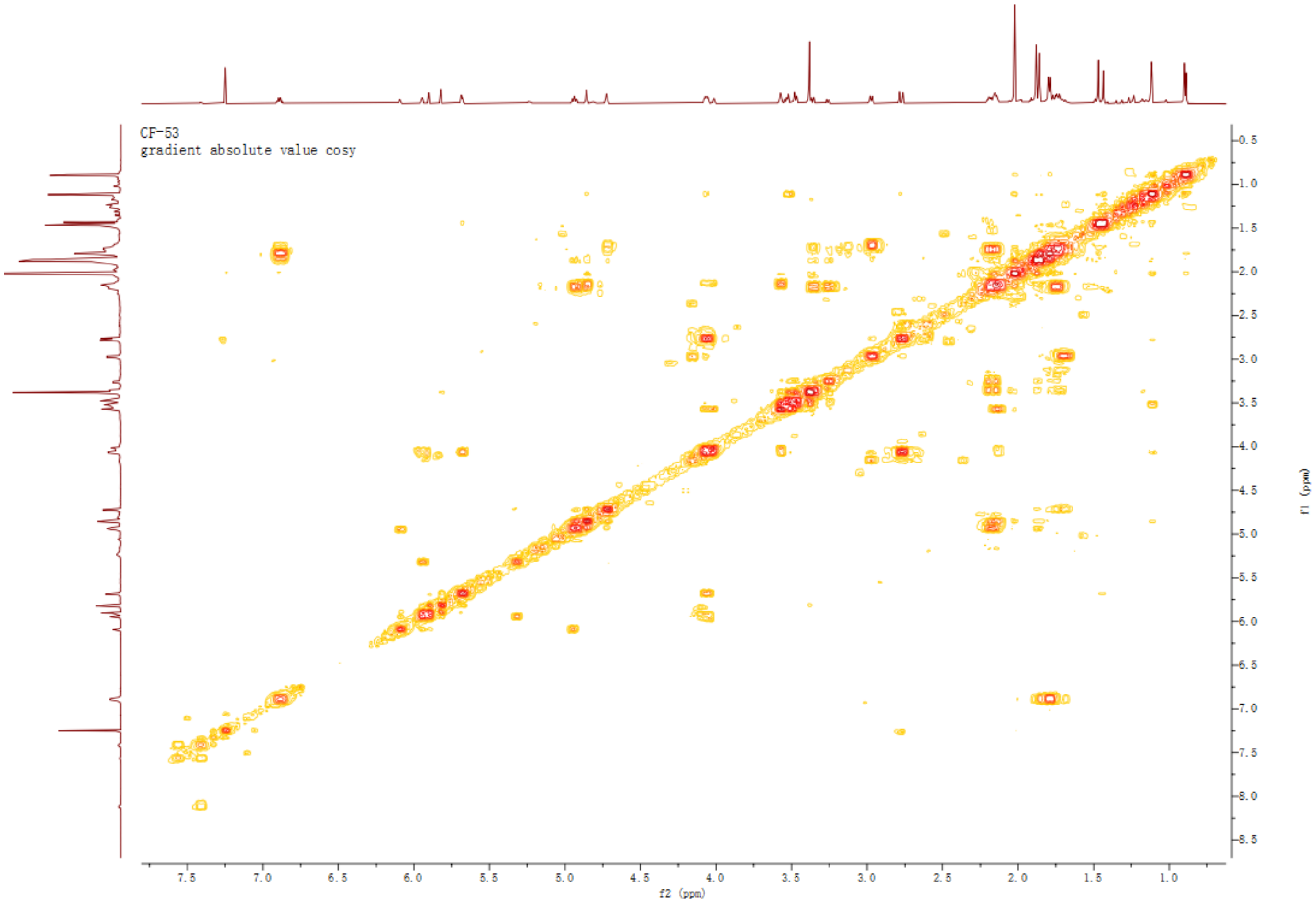

Figure S35. ^1H-^1H COSY spectrum of compound **5** in $CDCl_3$.

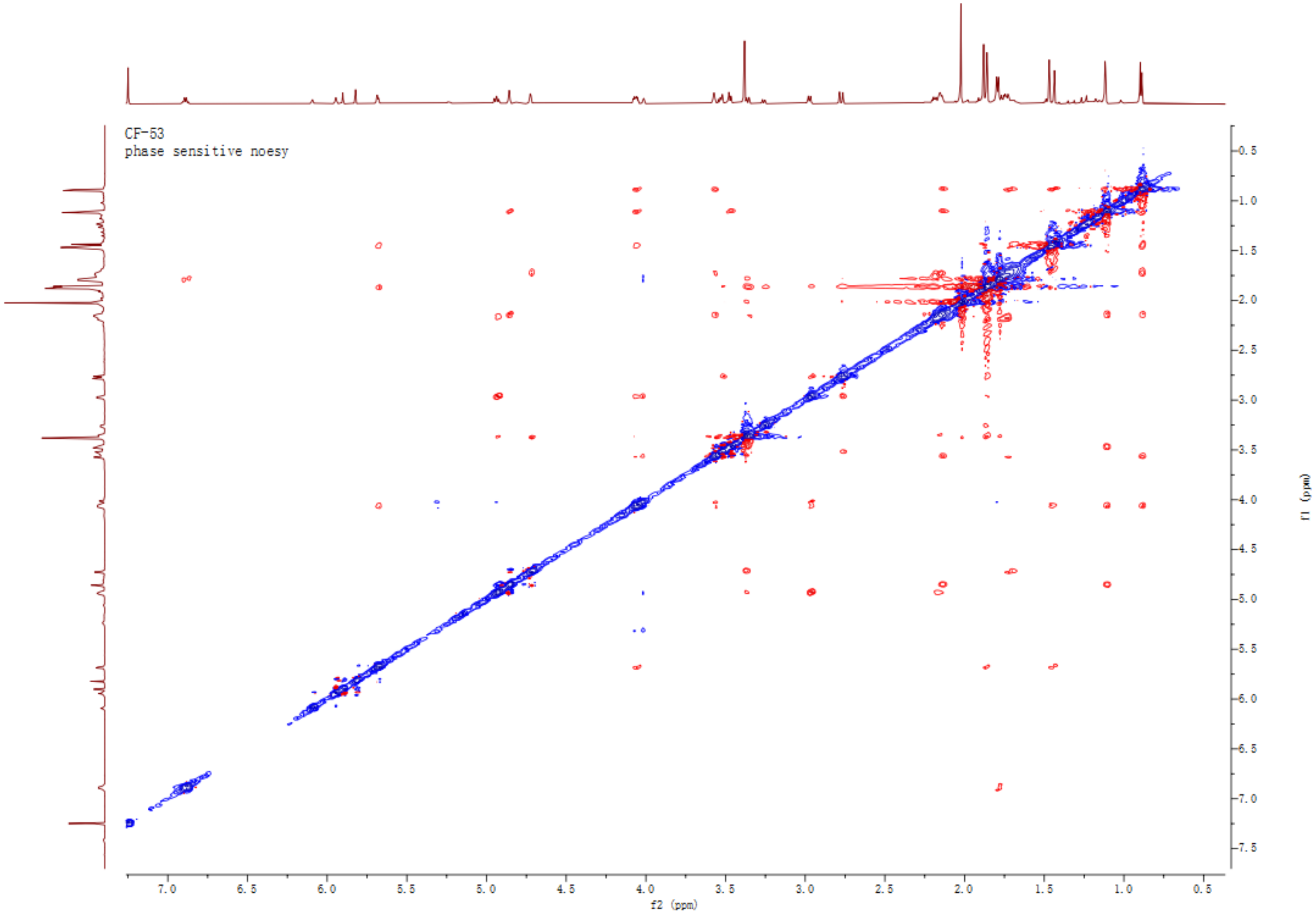

Figure S36. NOESY spectrum of Compound **5** in $CDCl_3$

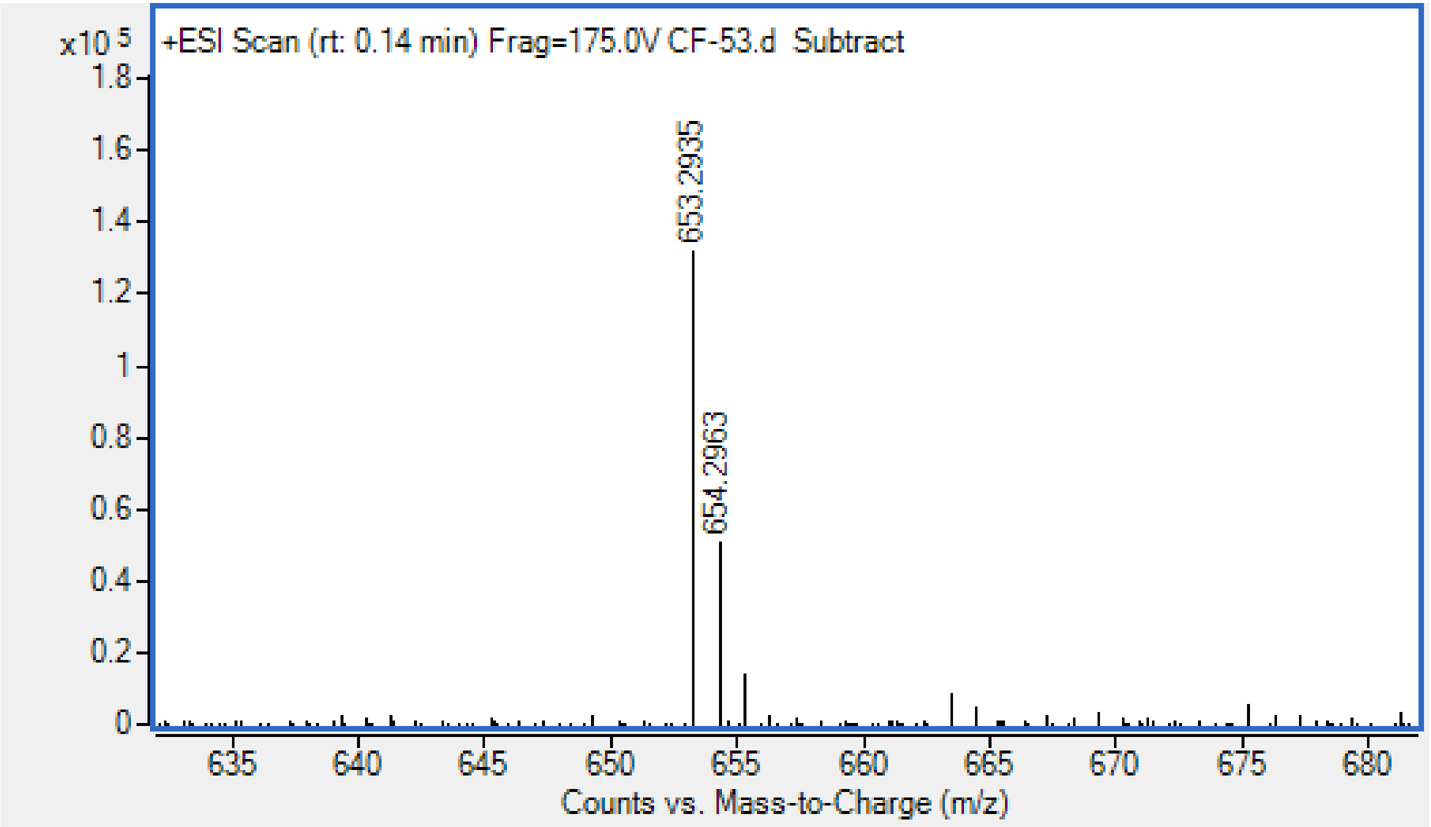

Figure S37. HRESIMS spectrum of compound **5**.
